# *MoPex19*, which Is Essential for Maintenance of Peroxisomal Structure and Woronin Bodies, Is Required for Metabolism and Development in the Rice Blast Fungus

**DOI:** 10.1371/journal.pone.0085252

**Published:** 2014-01-14

**Authors:** Ling Li, Jiaoyu Wang, Zhen Zhang, Yanli Wang, Maoxin Liu, Hua Jiang, Rongyao Chai, Xueqin Mao, Haiping Qiu, Fengquan Liu, Guochang Sun

**Affiliations:** 1 Department of Plant Pathology, College of Plant Protection, Nanjing Agricultural University, Nanjing, China; 2 State Key Laboratory Breeding Base for Zhejiang Sustainable Pest and Disease Control, Institute of Plant Protection Microbiology, Zhejiang Academy of Agricultural Sciences, Hangzhou, China; University of Nebraska, United States of America

## Abstract

Peroxisomes are present ubiquitously and make important contributions to cellular metabolism in eukaryotes. They play crucial roles in pathogenicity of plant fungal pathogens. The peroxisomal matrix proteins and peroxisomal membrane proteins (PMPs) are synthesized in the cytosol and imported post-translationally. Although the peroxisomal import machineries are generally conserved, some species-specific features were found in different types of organisms. In phytopathogenic fungi, the pathways of the matrix proteins have been elucidated, while the import machinery of PMPs remains obscure. Here, we report that *MoPEX19*, an ortholog of *ScPEX19*, was required for PMPs import and peroxisomal maintenance, and played crucial roles in metabolism and pathogenicity of the rice blast fungus *Magnaporthe oryzae. MoPEX19* was expressed in a low level and Mopex19p was distributed in the cytoplasm and newly formed peroxisomes. *MoPEX19* deletion led to mislocalization of peroxisomal membrane proteins (PMPs), as well peroxisomal matrix proteins. Peroxisomal structures were totally absent in Δ*mopex19* mutants and woronin bodies also vanished. Δ*mopex19* exhibited metabolic deficiency typical in peroxisomal disorders and also abnormality in glyoxylate cycle which was undetected in the known *mopex* mutants. The Δ*mopex19* mutants performed multiple disorders in fungal development and pathogenicity-related morphogenesis, and lost completely the pathogenicity on its hosts. These data demonstrate that *MoPEX19* plays crucial roles in maintenance of peroxisomal and peroxisome-derived structures and makes more contributions to fungal development and pathogenicity than the known *MoPEX* genes in the rice blast fungus.

## Introduction

Peroxisomes are ubiquitously present in eukaryotic cells, and typically consist of a protein-rich matrix surrounded by a single membrane. They are involved in various metabolic processes, such as H_2_O_2_ metabolism and fatty acid β-oxidation, and some organism-specific biochemical reactions, such as the synthesis of cholesterol, bile acids and plasminogen in mammals, the glyoxylate cycle in plants, and methanol oxidation in yeast [1]. In mammals, defects in peroxisomes result in a variety of developmental defects; the most severe of which is Zellweger syndrome, which can cause death in early childhood [2], [3].

The biogenesis of peroxisomes originates in the endoplasmic reticulum (ER) and consists of: (i) formation of the peroxisome membrane including acquisition of integral peroxisomal membrane proteins (PMPs); (ii) import of peroxisomal matrix proteins; and (iii) peroxisome proliferation [4]. Peroxisomes do not have their own DNA and therefore peroxisomal matrix proteins and PMPs are synthesized in the cytoplasm and imported into the organelles posttranslationally by complicated import machinery [5]. The proteins involved in peroxisomal import are designated as peroxins and their encoding genes are written as *PEX* [6]. The *PEX* genes were initially isolated in yeast models, and to date, more than 30 *PEX* genes have been identified in various organisms [1]. Homologues of most *PEX* genes are present in filamentous fungi [7]–[10].

Import of peroxisomal matrix proteins has been investigated intensively in different organisms, especially in yeast models. Most matrix proteins include a short peroxisomal-targeting signal (PTS), which can be either type PTS1 or PTS2 [4]. PTS1 is a tripeptide sequence (S/A/C) (H/R/K) (I/L/M) at the C terminus of the protein, and PTS2 is a nonapeptide sequence (R/K) (L/V/I) X5(H/Q) (L/A) at the N terminus. *PEX5* and *PEX7* encode receptors that recognize and bind PTS1- and PTS2-containing proteins respectively [11]–[14]. *PEX6* encodes an AAA type ATPase, and is involved in the import of peroxisomal matrix proteins by mediating the recycling of PTS receptors. In *Aspergillus nidulans*, *Magnaporthe oryzae* and *Fusarium graminearum*, *PEX6* mutation results in mislocalization of PTS1- and PTS2-containing proteins, suggesting the involvement of *PEX6* in both import pathways [10], [15], [16].

PMPs depend upon import pathways distinct to the PTS1- or PTS2-dependent import routes [17]. Most PMPs have one or more membrane protein-targeting signal (mPTS) consisting of a cluster of basic residues [18]–[20]. Pex19p is now known as both an import receptor and a chaperone for PMPs, which shuttles between the cytosol and peroxisomal membrane, binds and stabilizes newly synthesized PMPs in the cytosol, and is essential for PMP targeting and import [21], [22]. Biophysical data indicate that Pex19p consists of a folded C-terminal and a flexible N-terminal sequence [23]. The C-terminal CAAX motif of Pex19p is essential for its farnesylation and ability to bind PMPs [24].

Peroxisomes in filamentous fungi were first characterized cytochemically in 1971, and peroxisomal biogenesis has been investigated intensively in recent years [25]. Research on peroxisomal biogenesis in filamentous fungi has revealed distinct features that were undetected in yeasts or mammalian cells [9], [26]–[29]. An intriguing feature is their impact to pathogenicity of plant fungal pathogens. Disruption of *PEX6* gene (*ClaPEX6*) in anthracnose fungus *Colletotrichum lagenarium* (syn. *C. orbiculare*) damages peroxisomal metabolism and impairs infection severely, indicating the involvement of peroxisomal metabolism in pathogenicity of plant fungal pathogens [9]. This conclusion was reinforced by Δ*mgpex6* mutant in *M. oryzae*, which lacks appressorial melanization and fails in host penetration, and is therefore completely non-pathogenic [8], [30]. *PEX13* is required for appressorium-mediated plant infection by *C. orbiculare* [31]. *PEX5* and *PEX6* are critical to virulence and survival of *F. graminearum* on wheat [10]. In *M. oryzae*, the PTS1 pathway mediated by *MoPEX5* and PTS2 pathway mediated by *MoPEX7* are both required for fungal pathogenicity, although the PTS1 pathway seems to play a predominant role [32], [33]. Woronin bodies, which are fungus-specific organelles derived from peroxisomes, are also involved in pathogenicity of plant pathogens [34]. However, the characterized genes in fungal pathogens are all involved in the import of peroxisomal matrix proteins, whereas the import machinery of PMPs is still obscure.

*M. oryzae* is a heterothallic, haploid ascomycetous fungus that causes rice blast, the most destructive rice disease worldwide, and diseases on many other economically important cereal crops [35]. Infection with *M. oryzae* is initiated from the conidia, which are dispersed by splashing and tightly adhered to the hydrophobic leaf surface by an adhesive mucilage secreted on their tip [36]. Subsequently, the conidia germinate quickly and, within 8 h, form specialized infective structures called appressoria. The appressoria are darkly pigmented with melanin and accumulate high concentrations of glycerol to generate large internal turgor [37]. The appressoria attach to the leaf surface and immediately proceed with emergence of a slender hypha, the penetration peg, which ruptures the plant cuticle and cell wall to invade the underlying epidermal cells [38], [39]. During appressorial maturation and turgor generation, *M. oryzae* transfers abundant lipid bodies to the developing appressorium, coupled with rapid lipolysis. In eukaryotes, lipolysis requires the metabolic processes in peroxisomes [30]. End product of peroxisomal lipolysis, acetyl-CoA, is also a major source for infection related morphogenesis of *M. oryzae* [30].

To understand better the molecular mechanisms of PMP import and their roles in fungal pathogenesis, we characterized *MoPEX19*, the ortholog of *PEX19* gene in *M. oryzae*. Our data demonstrated that *MoPEX19* plays crucial roles in peroxisomal maintenance and pathogenicity of the rice blast fungus.

## Materials and Methods

### Fungal strains, growth conditions and transformation

*M. oryzae* wild-type Guy-11 [40] and all its derivative transformants and mutants strains were routinely cultured on complete medium (CM) [41] at 28°C for 3–14 days [42]. For genomic DNA isolation, mycelia were cultured in liquid CM for 3 days. Lipid medium, glucose medium and sodium acetate medium were prepared as described previously [33]. All fungal transformants were generated by *Agrobacterium tumefaciens*-mediated transformation (*At*MT) as described previously [43]. CM plates containing 250 μg/ml hygromycin B (Roche, Mannheim, Germany), 200 μg/ml glufosinate–ammonium (Sigma, St Louis, MO, USA) or 800 μg/ml G418 (Sigma) and defined complex medium (DCM; 0.16% yeast nitrogen base without amino acids, 0.2% asparagine, 0.1% ammonium nitrate and 1% glucose, pH 6.0 with Na_2_HPO_4_) [8] containing 100 μg/ml chlorimuron ethyl (Sigma) were used for screening corresponding transformants.

### Bioinformatic analysis

The homolog of *PEX19* in *M. oryzae* was identified by searching the *Magnaporthe* genome database (http://www.broadinstitute.org/annotation/genome/magnaporthe_comparative/MultiHome.html) with the protein sequence of Scpex19p from *Saccharomyces cerevisiae* (CAA98630.1). Sequence alignments were performed using the mafft program (http://mobyle.pasteur.fr/cgi-bin/portal.py#forms::mafft), with the parameters Gap opening penalty of 2.0, offset value of 0.1. The resulting alignments were imported into the software GeneDoc 2.0 for type setting and into MEGA 5.0 to establish phylogenetic trees.

### Nucleic acid manipulation and Southern blotting

The genomic DNAs were isolated using the cetyl trimethyl ammonium bromide method [41]. The restriction digestion, gel electrophoresis, ligation reactions, and PCR were all carried out using standard procedures. Southern blotting was performed using the digoxin high-prime DNA labeling and detection starter kit I (Roche) following the manufacturer's instructions.

Total RNA was isolated using the Trizol reagent (Invitrogen, Carlsbad, CA, USA), and used as a template to synthesize cDNA using AMV Reverse Transcriptase (Takara Bio, Otsu, Japan). The abundance of *MoPEX19* transcripts was analyzed using primer pair 19RTF/19RTR on the 7500 Fast Real-Time System (Applied Biosystems, Foster, CA, USA) by calculating the average threshold cycle (Ct) value, which was normalized to that of *Tubulin* gene (MGG_00604). The relative abundance of gene expression at deferent developmental stages was compared with that in fresh conidia. Three replicates were performed for each sample.

### Yeast complementation

The 1.1-kb fragment of *MoPEX19* cDNA was amplified using the primer pair 19pyes2cds4/19pyes2cds5 and inserted into the *Sma*I/*Bst*XI sites of the yeast expression vector pYES2 (Invitrogen) to generate pYES2-PEX19. pYES2-PEX19 was transformed respectively into *S. cerevisiae* wild-type strain BY4741 (MATa: *his3*Δ*1 leu2*Δ*0 met15*Δ*0 ura3*Δ*0*) and its derivative *ScPEX19* (YDL065C) deleted mutant (Thermo Scientific, San Jose, CA, USA) using the lithium acetate method. To assess the ability of the transformants to utilize oleic acid, the yeast cells were precultured in liquid YPD medium (1% yeast extract, 2% peptone and 2% dextrose) to mid-log phase, washed three times with sterilized double distilled water, adjusted to OD_600_ = 1, and then grown on YNO medium [0.67% yeast nitrogen base with amino acids (Sigma), 0.1% oleic acid and 0.05% Tween 40, adjusted to pH 6.0] and SD medium (0.67% yeast nitrogen base with amino acids and 2% dextrose, adjusted to pH 6.0) in 5-μl aliquots of 10-fold serial dilutions at 30°C for 13–15 h.

### *MoPEX19* deletion and mutant recovery

For gene deletion, hygromycin phosphotransferase (*HPH*) gene expression cassette was amplified with primer pair HPH-BamHI/HPH-SmaI and inserted into S*ma*I/B*am*HI sites of pCAMBIA1300 (CAMBIA, Canberra, ACT, Australia) to generate p1300-KO. A 1.6-kb *Pvu*I/*Xba*I upstream flanking sequence and 1.56-kb *Eco*RI/*Kpn*I downstream flanking sequence of *MoPEX19* were amplified from *M. oryzae* genomic DNA with primer pairs 19uf/19ur and 19df/19dr respectively, and inserted into p1300-KO to generate the disruption vector pKO-MoPEX19, which was introduced into the *M. oryzae* wild-type. The candidates of gene deletion mutant were selected from hygromycin-resistant transformants by genomic PCR with primer pair 19cds2/19cds3 and confirmed by Southern blotting. Two of the confirmed mutants, Δ*mopex19*-11 and Δ*mopex19*-44, were selected for phenotypic analysis.

For mutant recovery, a 3.5-kb fragment containing the full length of *MoPEX19* (1.2 kb), upstream (1.6 kb) and downstream (0.7 kb) sequences was amplified with primer pair 19comup/19comdn, and inserted into *Eco*RI/*Xba*I sites of p1300BAR [16] to generate complementary vector p1300BAR-19com, which was introduced into Δ*mopex19*-44. The resulting glufosinate–ammonium-resistant transformants were picked up, from which the complemented transformants were selected by genomic PCR with primer pair 19cds2/19cds3 and confirmed by detecting the gene transcripts using quantitative PCR with primer pair 9RTF/19RTR. One of the confirmed transformants, Δ*mopex19*-com, was used in phenotypic analysis.

### Generation of fluorescent protein fusion constructs

To monitor the gene expression of *MoPEX19*, the 1.5-kb promoter region upstream *MoPEX19* open reading frame (ORF) was amplified using the primers 19proup and 19prodn, and inserted into pBP5GFP [33] by *Pvu*I/*Xba*I digestion to substitute the *MoPEX5* promoter and generate the GFP expression vector p1300BP19GFP.

To track the cellular localization of *MoPEX19*, the coding sequence of GFP without termination codon was amplified with the primer pair GFP-Xb/GFP-C-Sm, and introduced into p1300BMGFP [16] by *Xba*I/*Sma*I digestion to replace the intact GFP ORF and produce p1300BMGFP-C. The coding sequence of *MoPEX19* was amplified using the primer 19cds2/19cds3 from the total RNA as template, and introduced into p1300BMGFP-C by *Sma*I/*Bst*XI digestion to produce the fusion vector p1300BMGFP-PEX19, in which the GFP–MoPEX19 fusion could be expressed under the control of the *MPG1* promoter [41]. To mark the position of the peroxisomes, the fusion vector p1300NMRFPA containing RFP–PTS1 [16] was co-integrated into the p1300BMGFP-PEX19 transformants.

To investigate the localization of PTS1- and PTS2-containing proteins in wild-type, Δ*mopex19* mutant and complemented strains, the p1300BMGFPA and p1300BMGFPB [16] were modified by replacing their hygromycin resistance gene cassette with the neomycin phosphotransferase II cassette to generate p1300NMGFPA and p1300NMGFPB, respectively. The ortholog of *PMP47* (MGG_03247, assigned as *MoPMP47* in present work) was used as a representative to assess the localization of PMPs. The MGG_03247 ORF was amplified with the primers P47-cds2 and P47-cds3, and introduced into p1300SMGFP-C, a vector derived from p1300BMGFP-C by replacing its *BAR* gene cassette with chlorimuron ethyl resistance gene (*SUR*), to produce the fusion vector p1300SMGFP-PMP47.

The woronin body major protein, Hex1 (MGG_ 02696), was fused with RFP to mark woronin bodies [34]. The coding sequence of RFP without termination codon was amplified with the primer pair RFP-Xb/RFP-C-Sm from p1300NMRFPA [16], and introduced into p1300NMRFPA by *Xba*I/*Sma*I digestion to produce p1300NMRFP-C. The coding sequence of *MoHEX1* was amplified using the primer HexC-54/HexC-31-BstXI, and introduced into p1300NMRFP-C by *Sma*I/*Bst*XI digestion to produce p1300NMRFP-HEX1 [41]. All the primers used in present work were listed in Table 1.

**Table 1:** The primers used in this study.

Name	Sequence (5′–3′)
19RTF	GAGGAACAAGGACAAGACTTC
19RTR	CATCTTTTGCATCCTATCCA
19pyes2cds4	CCCAAGCTTAACAAAATGGCGGACCGGCCCGAGGACACA
19pyes2cds5	GCTCTAGAATAGCATTTTGGCATTGATAGTCA
HPH-BamHI	CGGGATCCTGGAGGTCAACACATCAATGCTATT
HPH-SmaI	CCCCCGGGCTACTCTATTCCTTTGCCCTCGGAC
19uf	AACTGCAGTTGAAAAGGTTGTATGCGTCTGCC
19ur	GCTCTAGATGGAGGAAAACCCCTGGAATACTT
19df	GGGGTACCAAAACGCCATAACCTAATCCCACC
19dr	CGGAATTCGCCTAGTAATGTCTAAGAAGCCAACA
19cds2	TCCCCCGGGATGGCGGACCGGCCCGAGGACACA
19cds3	CTGCAGAACCACCATGTTGGGAACATGCTCTCATTTTGTCGTTT
19comup	CGGAATTCCGCTGGACGCCGATGAGGATCTGCTTGA
19comdn	GCTCTAGAGGAGAGGAGAGGTGTCGAGCACAAG
19proup	ATCGATCGTTGAAAAGGTTGTATGCGTCTGCC
19prodn	GCTCTAGAGGCCCTGACTTTTTGCTAGGTCGT
GFP-Xb	GCCCTCTAGAATGGTGAGCAAGGGCGAGGA
GFP-C-Sm	TCCCCGGGCTTGTACAGCTCGTCCATGCCGAG
NEO-Xh1	CCGCTCGAGGAGGTCAACACATCAATGC
NEO-Xh2	CCGCTCGAGTCAGAAGAACTCGTCAAGAAGGCG
SUR-Xh1	CCGCTCGAGGTGCCAACGCCACAGTGCC
SUR-Xh2	CCGCTCGAGGTGAGAGCATGCAATTCCC
P47-cds2	TCCCCCGGGTTGGTTGCCGCTTCGAAGCCCGTC
P47-cds3	CGGAATTCTATTTCTAAACTTGACAATCAAAG
HexC-54	TCCCCCGGGTTGGGTTACTACGAAGACGACCGT
HexC-31-BstXI	CTGCAGAACCAATGCATTGGCATCTAGGTTTAGAGCTAGAGAGT

The restriction sites used were underlined.

### Analysis of conidial germination, appressorial formation, turgor pressure and penetration

Conidia harvested from 10-day-old CM plates were suspended at 1×10^5^/ml. Aliquots (30 μl) of the suspensions were incubated on a plastic coverslip in a moist chamber at 28°C for 48 h. Conidial germination and appressorium formation were examined at 2, 4, 6, 8, 12, 24, 48 h post-incubation.

The incipient-cytorrhysis assay was performed to measure appressorial turgor. Conidia in 20 μl droplets (2×10^5^/ml) were allowed to form appressoria on plastic coverslips for 48 h. Water surrounding the conidia was removed carefully and replaced with 20 μl 0.5–4.0 M glycerol. The number of collapsed appressoria was counted after 10 min. The experiments were replicated three times, and >200 appressoria were observed in each.

### Tolerance to H_2_O_2_, methyl viologen and Congo red

To assess the vegetative growth under oxidative stress, 2.5 or 5.0 mM H_2_O_2_ or 1 mM methyl viologen were added to CM, and the colonial diameters cultured for 3–5 days were measured. Cell wall integrity was assayed by growing the strains on CM supplemented with 200 μg/ml Congo red for 5 days.

### Pathogenicity tests and infectious structures observation

Two-week-old rice CO39 seedlings and 7-day-old barley ZJ-8 were used for pathogenicity tests with conidia harvested from 10-day-old CM plates. Suspensions of 2×10^4^ conidia/ml supplemented with 0.25% (w/v) gelatin were applied by spray inoculation as described previously [41]. In the inoculation on detached leaves, the 30-μl droplets of the conidial suspension or mycelial plugs (5 mm diameter) were placed on leaf sections, and the leaves were incubated at 28°C in darkness for 24 h and then in light for 3 days. For inoculation of wounded leaves, the detached barley leaves were scraped with sandpaper to remove the cuticles. To observe infectious structures, the inoculated barley leaves were discolored with lactic acid, heated at 65°C for 2 h, and examined microscopically.

### Fluorescent microscopy and transmission electron microscopy (TEM)

The cellular localization of GFP and red fluorescent protein (RFP) fusions were detected under a Leica SP2 Confocal System (Mannheim, Germany), with excitation 488 nm, emission 520 nm for GFP and excitation 558 nm, emission 583 nm for RFP. Nile red staining and fluorescein diacetate (FDA) staining were performed as previously described [33], and the fluorescence was observed under an Olympus Xa21 fluorescent microscope with filters adapted to excitation 495 nm and emission 520 nm. (Tokyo, Japan).

For TEM analysis, the conidia and mycelia were collected on CM plate cultured at 28°C for 3–14 days. The collected fungal mass was treated as described [44] and examined under a JEM-1230 electron microscope (JEOL, Tokyo, Japan).

## Results

### Identification of *MoPEX19*

**Figure 1:**
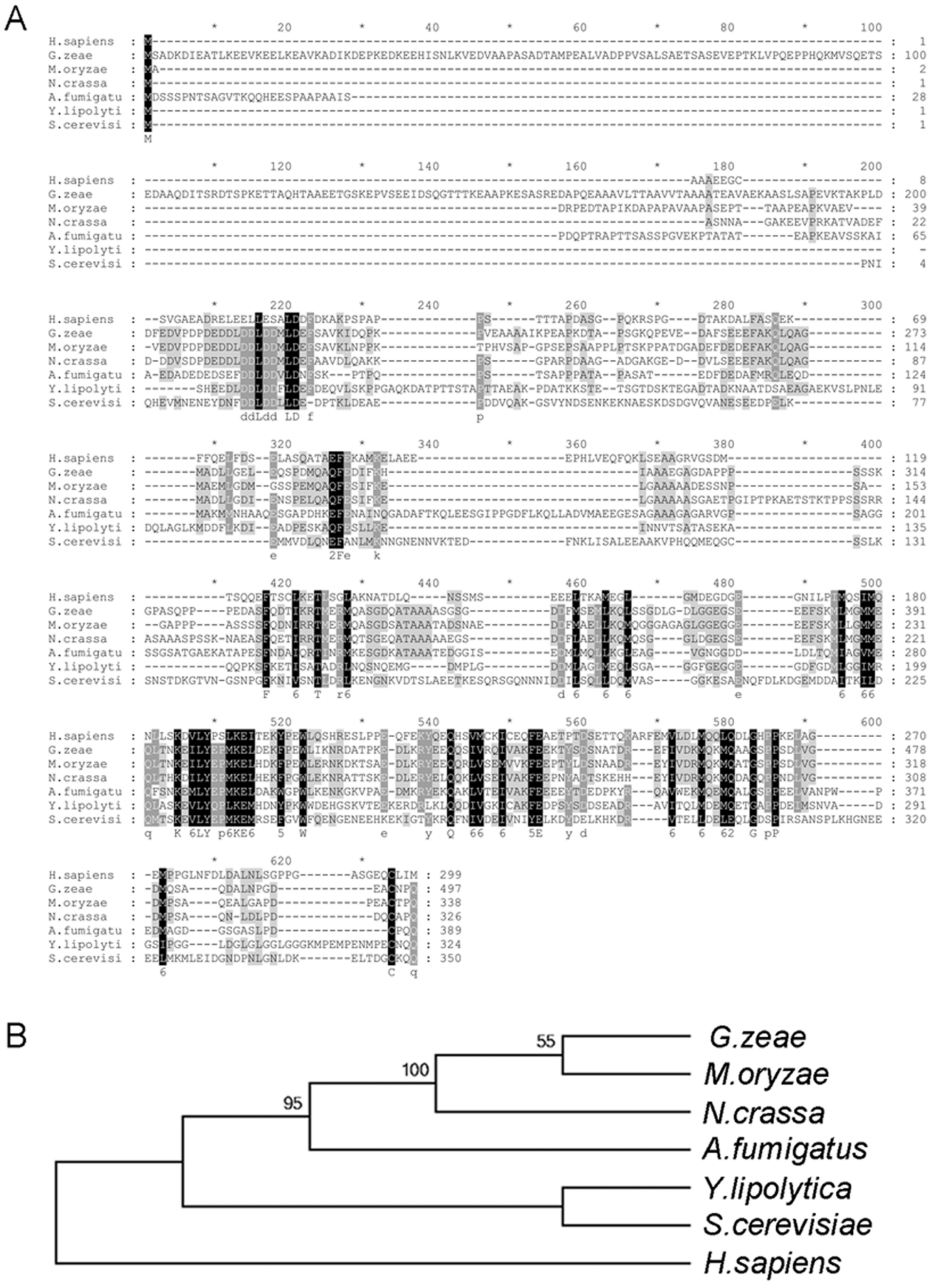
Similarity of *MoPEX19* homologs. (A) Amino acid sequences of *HsPex19* (XP_501231.1) from humans, *GzPex19* (XP_390112.1) from *Gibberella zeae* (*F. graminearum*), *NcPex19* (XP_961091.1) from *N. crassa*, *AfPex19* (XP_754525.1) from *Aspergillus fumigatus*, and *ScPex19* (CAA98630) from *S. cerevisiae* were aligned with ClustalW. Identical amino acids are highlighted against a black background, conserved residues are shown on a dark gray background, and similar residues on a light gray background. (B) Phylogenetic relationship of *PEX19* homologs calculated with neighbor-joining method using the MEGA 5.0 program according to alignment.

To find the *PEX19* homologs in *M. oryzae*, the 350-amino-acid protein sequence of *S. cerevisiae* Scpex19p (CAA98630.1) was used to search the *Magnaporthe* comparative database with the BlastP procedure. A hypothetical gene MGG_00971 exhibited the most similarity to Scpex19p, with 24% amino acid identity. cDNA sequencing confirmed that the ORF of MGG_00971 was 1221-bp long, with three introns and four exons, and encoded a polypeptide of 356 amino acid residues, which was completely consistent with the annotation in the genome database. The predicted protein sequence of MGG_00971 showed 62% amino acid identity to hypothetical Pex19p from *Gibberella zeae* (XP_390112.1) and 57% identity to hypothetic Pex19p from *Neurospora crassa* (XP_961091.1) (Fig. 1). MGG_00971 was thus regarded as the *PEX19* homolog in *M. oryzae*, and assigned as *MoPEX19*.

### *MoPEX19* is a functional homolog of *PEX19* in *S. cerevisiae*

**Figure 2:**
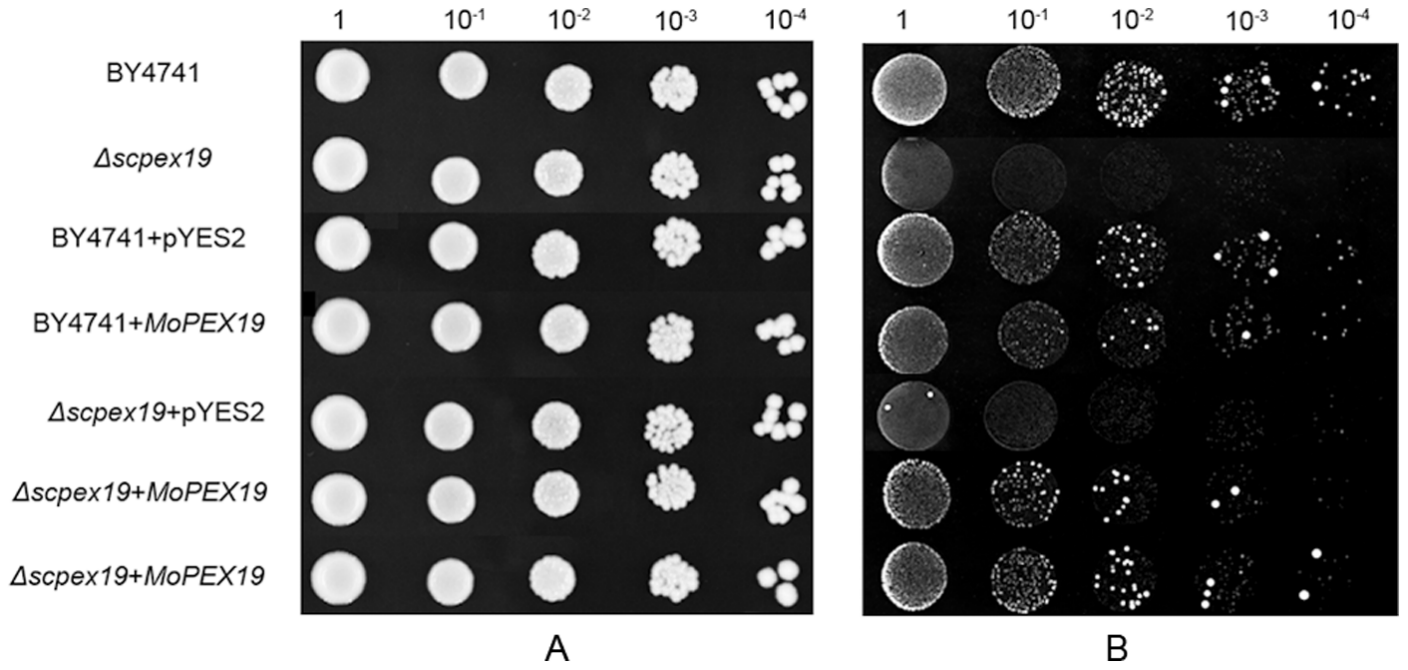
Complementation of Δ*scpex19* mutant by *MoPEX19*. *S. cerevisiae* wild-type (BY4741), Δ*scpex19*, and transformants BY4741+pYES2, BY4741+*MoEX19*, Δ*scpex19*+pYES2 and Δ*scpex19*+*MoPEX19* were cultured on SD plates with glucose (A) or oleic acid (B). The expression of *MoPEX19* restored the ability of Δ*scpex19* to grow on oleic acid.

To investigate whether *MoPEX19* (MGG_00971) is a functional ortholog of *PEX19*, we tested the ability of *MoPEX19* to complement *S. cerevisiae pex19* mutant. *MoPEX19* cDNA carried by pYES2 was introduced into Δ*scpex19* mutant derived from strain BY4741. Δ*scpex19* exhibited severe growth deficiency on oleic acid medium, while the *MoPEX19* integration fully restored the growth of the mutant on oleic acid (Fig. 2), suggesting that *MoPEX19* has a function similar to *ScPEX19*.

### *MoPEX19* was distributed in cytoplasm and newly formed peroxisomes

**Figure 3:**
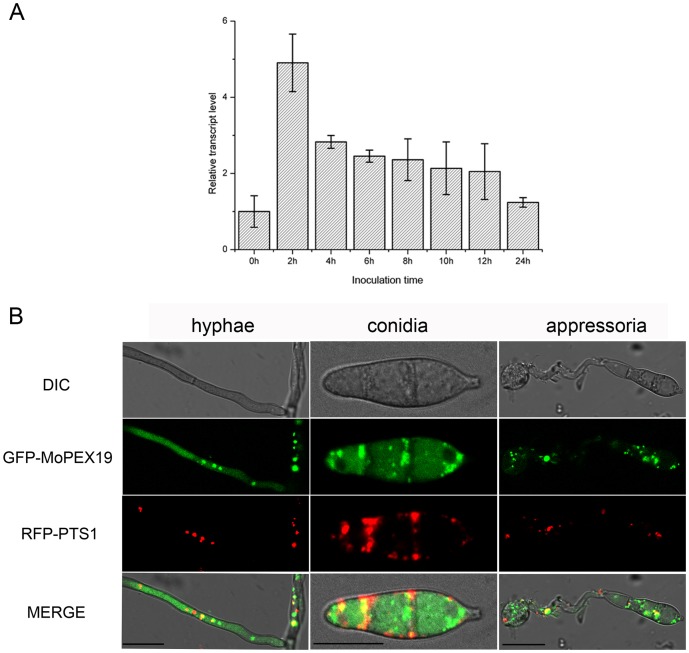
Sequential expression and cellular localization of *MoPEX19*. (A) Relative transcript abundance of *MoPEX19* during appressorial development. Transcript abundance normalized to β-tubulin gene was measured by quantitative PCR at each time point and compared with that in the non-incubated conidia. (B) Fluorescent microscopy of co-transformants with GFP–MoPEX19 and RFP–PTS1 in hyphae, conidia and appressoria of *Magnaporthe oryzae*. The fluorescence of GFP–MoPEX19 was predominantly in the cytoplasm with some punctate enhancement, which partially overlapped with the red fluorescence of RFP–PTS1. Bar = 10 μm.

The abundance of *MoPEX19* transcripts was analyzed by quantitative PCR, which showed that the transcription of *MoPEX19* was upregulated during conidial germination and appressorial development, with a peak at the initial germinated conidia incubated for 2 h (Fig. 3A). We examined the expression of GFP under the *MoPEX19* promoter, but no fluorescence was visible in hyphae, conidia or appressoria, indicating that *MoPEX19* was expressed at a low level and its promoter was weak. To assess the subcellular distribution of Mopex19 protein, we analyzed the localization of GFP–MoPEX19 fusion controlled by the *MPG1* promoter. Fluorescent microscopy showed that the green fluorescence of GFP–MoPEX19 in hyphae, conidia or appressoria was present predominantly in the cytoplasm and enhanced at small puncta (Fig. 3B). The puncta overlapped partially with the fluorescence of RFP–PTS1, which represented the location of peroxisomes. *PEX19* was previously demonstrated functioning in initial stages of peroxisomal biogenesis before the import of matrix proteins [24]. We thus conclude that Mopex19p was localized in cytoplasm and newly formed peroxisomes.

### Disruption of *MoPEX19*

**Figure 4:**
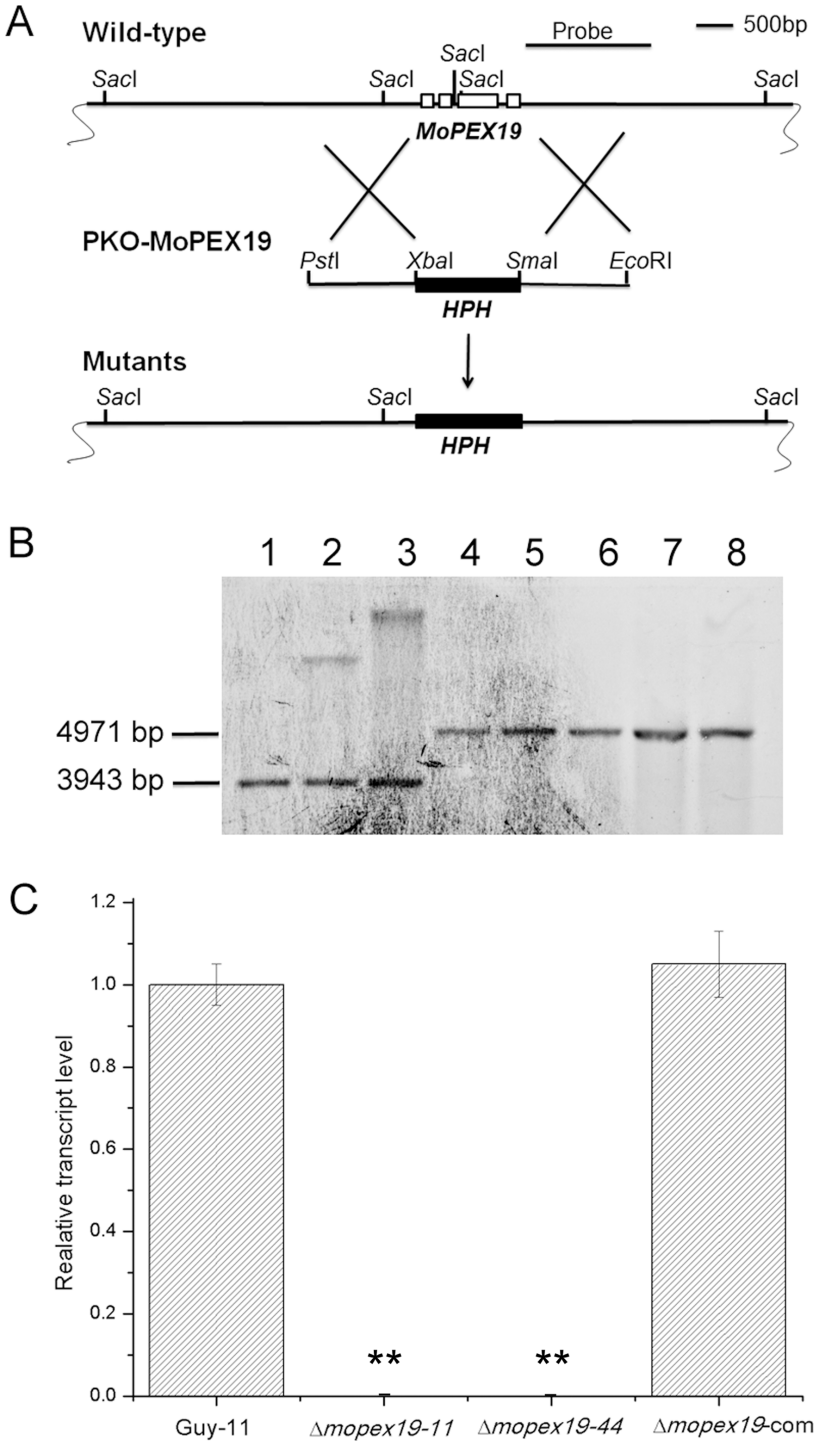
*MoPEX19* gene deletion and mutant complementation. (A) Diagram showing that the 1.22-kb *MoPEX19* coding region was replaced by the 1.36-kb *HPH* cassette. An inner fragment within the deletion region was used as the probe for Southern blotting. Scale bar = 500 bp. (B) Total genomic DNA was isolated from the wild-type (lane 1), ectopic transformants (lanes 2 and 3), and potential Δ*mopex19* mutants (lanes 4–8, Δ*mopex19-11*, Δ*mopex19-14*, Δ*mopex19-20*, Δ*mopex19-32* and Δ*mopex19-44*), digested with *Sac*I and subjected to Southern blotting. A 3943-bp hybridization band was detected in the wild-type, whereas 4971-bp bands were present in the five potential mutants, consistent with the gene deletion events. Ectopic transformant generated two bands, one of which was in equal size to the wild-type. (C) Gene transcription analysis of wild-type (Guy-11), Δ*mopex19-11* and Δ*mopex19-44*, and Δ*mopex19-*com by quantitative PCR. *MoPEX19* transcripts were detected in similar abundance in the wild-type and complemented strains, but were completely undetectable in the mutants.

To determine the roles of *MoPEX19* in peroxisomal biogenesis and fungal pathogenicity in *M. oryzae*, targeted gene replacement was performed. The knockout vector pKO-MoPEX19 was introduced into wild-type strain Guy-11 (Fig. 4A). Fifty hygromycin-resistant transformants were selected and checked primarily by PCR. Based on the PCR results, five of the possible gene-deleted mutants and two random insertion transformants were selected randomly and confirmed by Southern blotting (Fig. 4B), which indicated that gene replacement events occurred truly in the five mutants. Two of the confirmed mutants, Δ*mopex19-11* and Δ*mopex19-44*, were further tested by quantitative PCR, which ensured that the gene expression was completely removed (Fig. 4C). For mutant complementation, a genomic fragment containing full-length *MoPEX19* was reintroduced into Δ*mopex19-44*. The resulting transformants were selected primarily by genomic PCR, and potential complemented transformants of them were confirmed by checking the gene transcription (Fig. 4C). The two Δ*mopex19* mutants (Δ*mopex19-11* and Δ*mopex19-44)* and one of the confirmed complemented transformants (Δ*mopex19-*com) were used for phenotypic analysis.

### Peroxisomal matrix proteins and PMPs were mislocalized in *MoPEX19* deleted mutants

**Figure 5:**
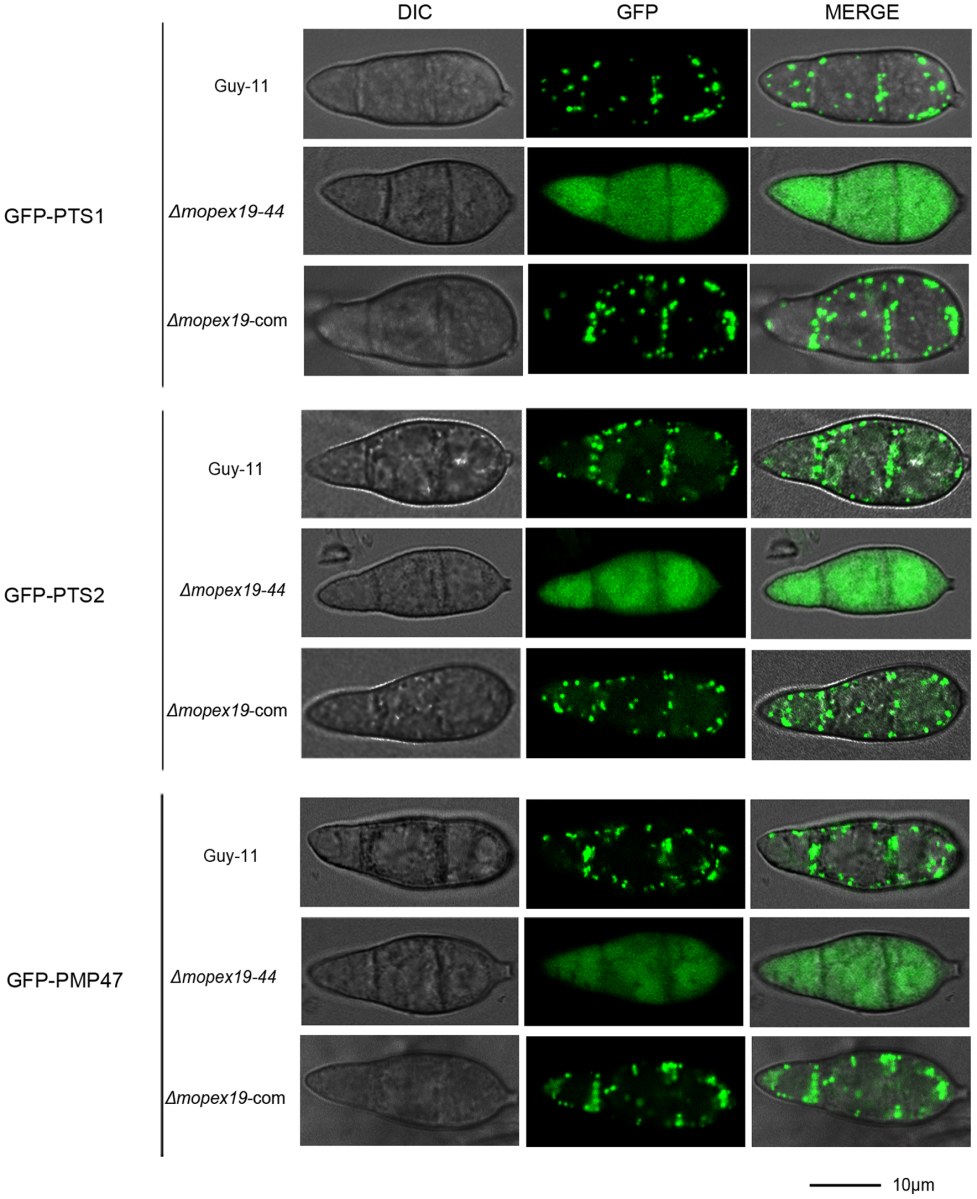
Distribution of the peroxisomal matrix proteins and PMPs in Δ*mopex19* mutants. Wild-type, Δ*mopex19-44* and complemented strains were transformed with GFP–PTS1, GFP–PTS2 and GFP–PMP47, respectively. Conidia of the transformants were harvested from 5-day-old CM plates and detected using confocal fluorescence microscopy. In the wild-type, the GFP–PTS1, GFP–PTS2 and GFP–PMP47 were all observed in punctate pattern, whereas in Δ*mopex19*, GFP fluorescence was dispersed in the cytoplasm. In the complemented strains, GFP fluorescence was recovered into punctate patterns. Bar = 10 μm.

To investigate the functions of *MoPEX19* in the import of the peroxisomal matrix proteins and PMPs, we assayed the cellular distribution of PTS1- and PTS2-containing proteins and Mopmp47 (representing the PMPs) in Δ*mopex19* mutants. The GFP fusions GFP–PTS1, GFP–PTS2 and GFP–PMP47 were introduced into the wild-type Guy-11, Δ*mopex19* mutants (Δ*mopex19-44*) and complemented strain (Δ*mopex19*-com), respectively and detected by laser scanning confocal microscopy. In the transformants derived from the wild-type, the GFP fusions with PTS1, PTS2 or Mopmp47 were all distributed in punctate patterns, indicating their proper peroxisomal localization. However, the localization patterns were totally changed in Δ*mopex19-44*, where GFP–PTS1, GFP–PTS2 and GFP–PMP47 were all distributed in the cytoplasm, suggesting that these proteins were unable to be imported into peroxisomes. The peroxisomal localization of the GFP fusions was recovered by reintroduction of *MoPEX19* (Fig. 5). These results indicate that *MoPEX19* is indispensable for the import of peroxisomal matrix proteins and PMPs into peroxisomes of *M. oryzae*.

### Peroxisomal structures and woronin bodies vanished in *MoPEX19* deleted mutants

**Figure 6:**
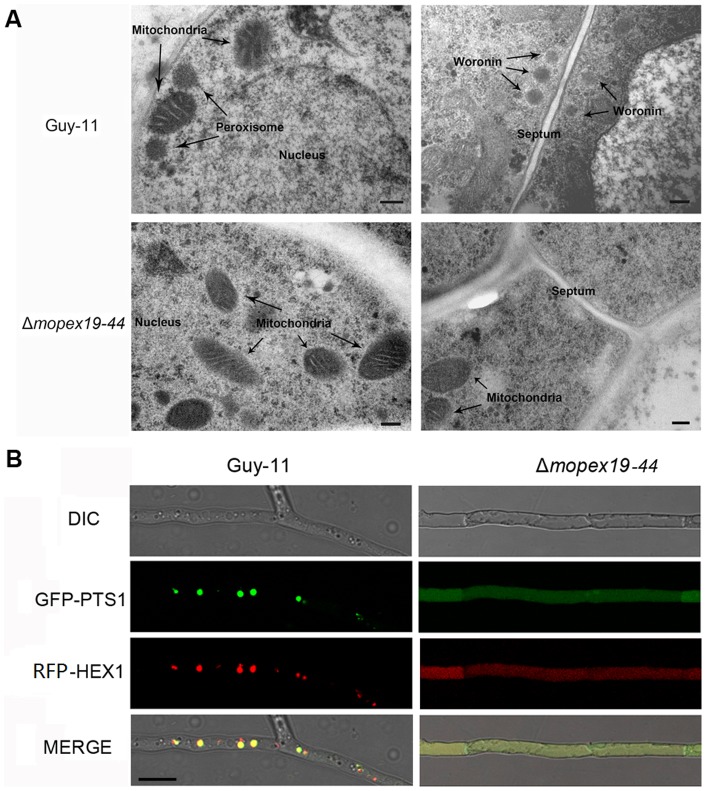
Peroxisome and woronin bodies were absent in Δ*mopex19* mutants. (A) Ultrastructure of the wild-type and Δ*mopex19* mutant. Hyphae and conidia from 7-day-old CM plates were analyzed by TEM. Peroxisomes, nuclei and mitochondria were detected in the wild-type (upper left), while the peroxisomes were absent in Δ*mopex19* (lower left). Typically 3–5 woronin bodies were seen beside the intercellular septum in the wild-type (upper right), but were undetectable in Δ*mopex19* (lower right). Bar = 0.2 μm. (B) Fluorescent localization of GFP-PTS1 and RFP-HEX1 in the wild-type and Δ*mopex19* mutant. Bar = 10 μm.

To investigate the influence of *MoPEX19* deletion on peroxisomal structures, we analyzed the ultrastructure of the Δ*mopex19* and wild-type by TEM. In the wild-type cells, round peroxisomes were detected, mainly in the periphery of the cells and differentiable to the mitochondria in shapes and sizes (Fig. 6). However, peroxisomes or peroxisome-like structures were not detectable in the Δ*mopex19-44* mutant. These results indicated that *MoPEX19* was vital to peroxisome maintenance. We also found that woronin bodies were absent in the Δ*mopex19* mutant, in contrast with the wild-type, in which they presented mainly adjacent to hyphal septa and with a smaller size than the peroxisomes (Fig. 6A). Further, fusion of RFP with the Woronin body protein Hex1 resulted in punctate distribution that predominantly overlapped with GFP–PTS1 in accordance with the expected position of Woronin bodies in wild-type hyphae [34]. In contrast, RFP–Hex1 and GFP–PTS1 were both dispersed in the cytoplasm of the Δmopex19 mutant (Fig. 6B). These data together indicated that the peroxisomes and woronins were absent in Δ*mopex19* mutant.

### *MoPEX19* deletion led to defects in lipid metabolism and tolerance of reactive oxygen species (ROS)

**Figure 7:**
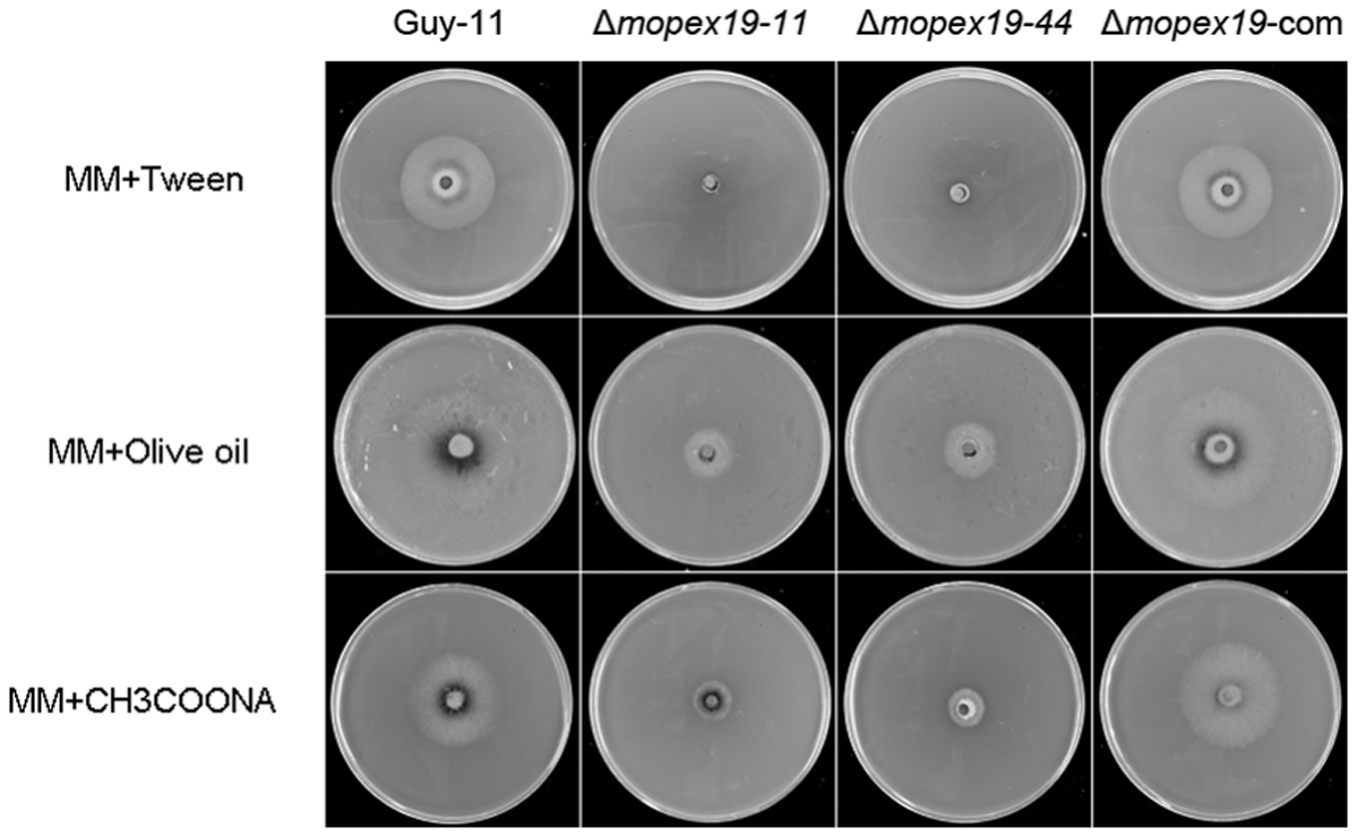
Growth tests to determine the ability of Δ*mopex19* to utilize lipids as sole carbon source. Wild-type, Δ*mopex19* and complemented strains were inoculated with a plug of mycelium on minimal medium (MM) supplemented with 0.5% (v/v) Tween 80, olive oil or 12 g/l sodium acetate and cultured at 28°C for 12 days.

**Figure 8:**
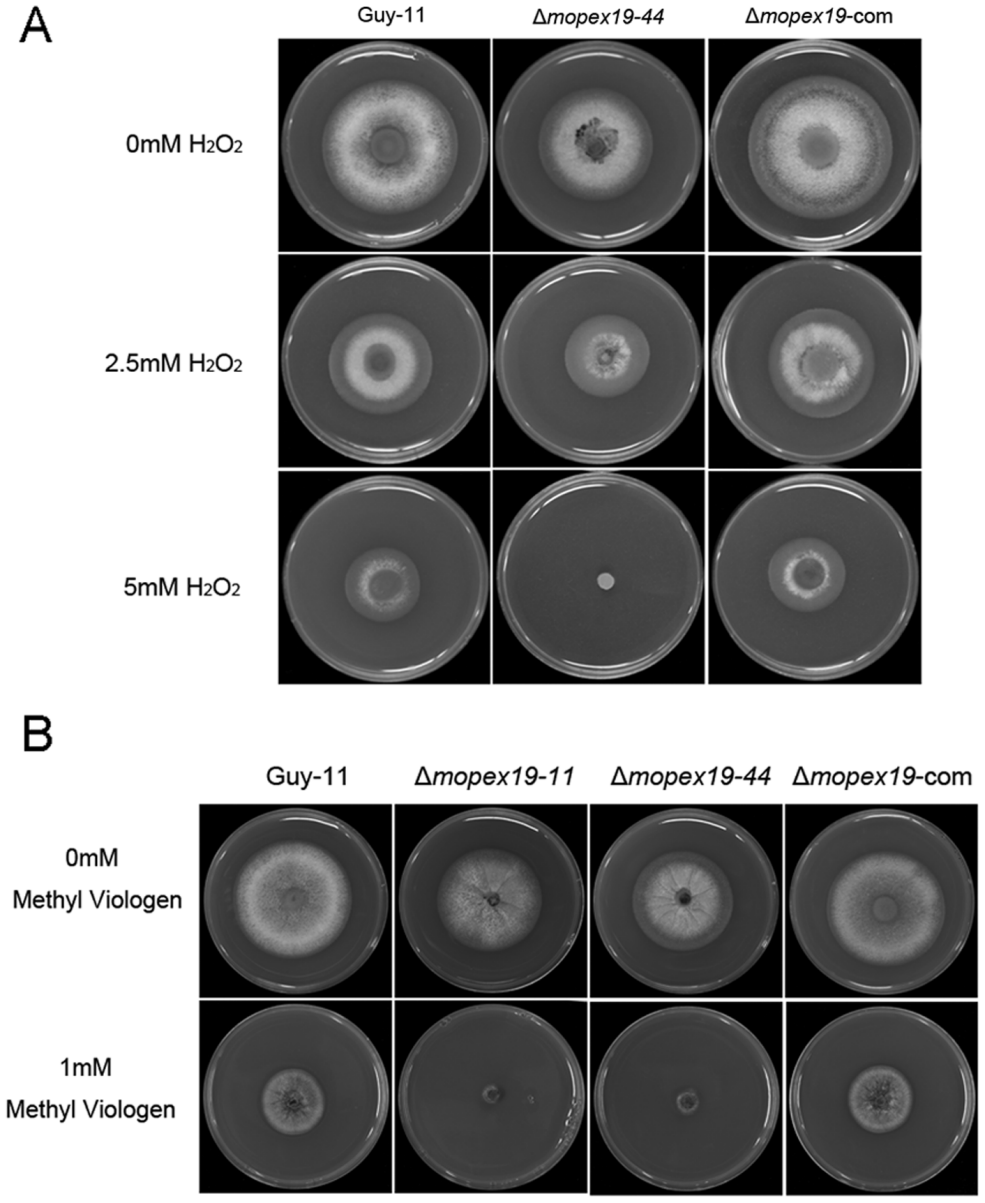
Tolerance of Δ*mopex19* to H_2_O_2_ and methyl viologen. Wild-type, Δ*mopex19* and complemented strains were cultured on CM and CM supplemented with H_2_O_2_ (A) or methyl viologen (B) at 28°C for 5 days.

To assess the effects of *MoPEX19* deletion to peroxisomal metabolism, we investigated the capacity of lipid utilization and ROS elimination of the mutants. On media with Tween 80, olive oil and sodium acetate as sole carbon source, the development of Δ*mopex19* mutants was decreased greatly compared with that of the wild-type and the complemented strain, indicating disruption of lipid metabolism in the mutants (Fig. 7). The capacity to eliminate ROS was examined by comparing the tolerance to H_2_O_2_ and methyl viologen. On media containing H_2_O_2_ or methyl viologen, mutant growth was significantly reduced compared with the wild-type and complemented strain (Fig. 8A and 8B). The results suggested that deletion of *MoPEX19* affected lipid metabolism and resistance to ROS in *M. oryzae*.

### Deletion of *MoPEX19* suppressed aerial hyphal growth and conidiation

**Figure 9:**
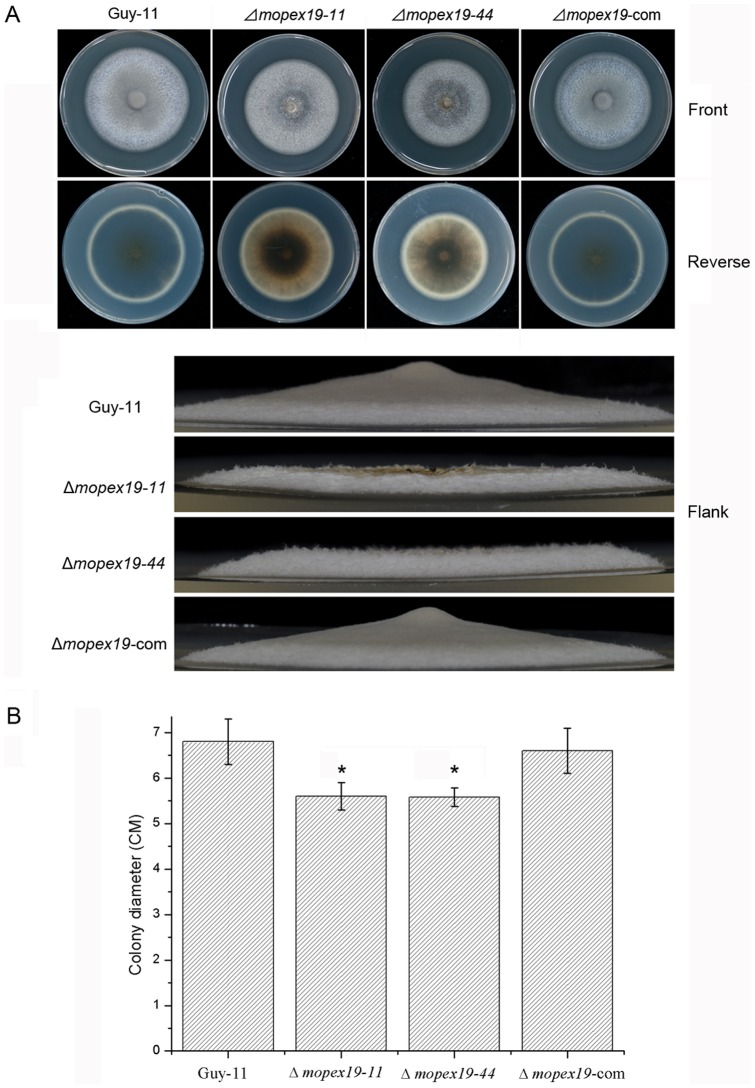
Vegetative growth of wild-type, Δ*mopex19* mutants and complemented strains. (A) The strains were cultured on CM at 28°C for 10 days. The radical growth, aerial hyphae growth and colonial pigmentation were decreased in Δ*mopex19* strains. (B) Measurement and statistic comparison of radial growth of the strains. Means and standard errors were calculated from three independent repeats. Single asterisks indicate significant differences to the wild-type (P<0.05).

The Δ*mopex19* mutants showed reduced radial growth and aerial hyphal development. On CM, Δ*mopex19-11* and Δ*mopex19-*44 formed flat and bald colonies, which were significantly smaller than those of the wild-type and complemented strains (Fig. 9). The colonial pigmentation of the mutants was also decreased compared with the wild-type and complemented strains. Along with the increase in culture time, cell death was seen in Δ*mopex19* mutants, which initiated from the colonial center and expanded towards the edge. This suggests that *MoPEX19* is essential for the vegetative growth and colonial melanization in *M. oryzae*.

**Figure 10:**
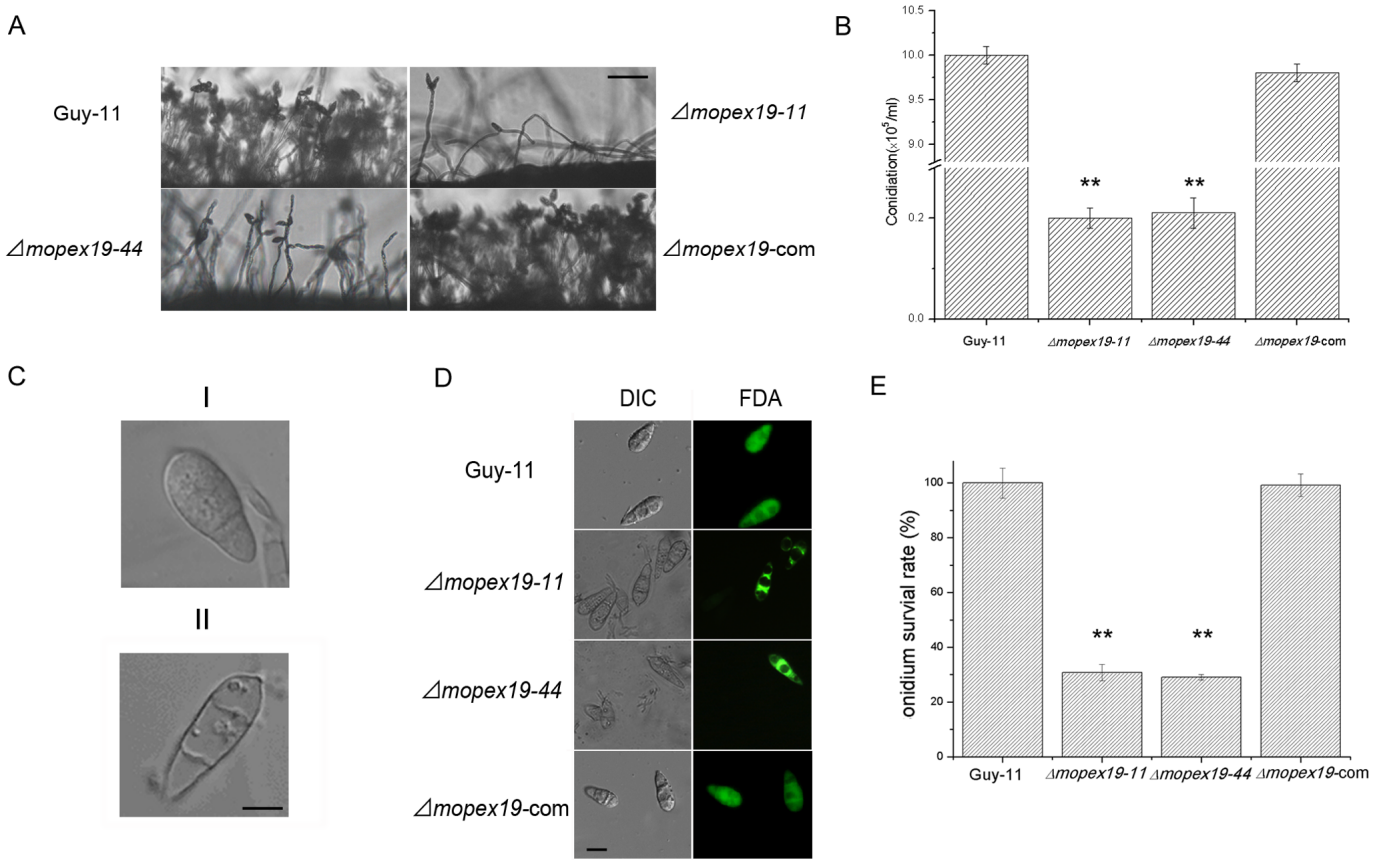
*MoPEX19* deletion reduced the conidiation and conidial morphology and viability. (A) Wild-type, Δ*mopex19* and complemented strains cultured on CM for 7 days were examined by light microscopy. Bar = 50 μm. (B) Statistical analysis of conidia produced on CM culture at 28°C for 10 days. Means and standard errors were calculated from three independent repeats. Double asterisks indicate significant differences to the wild-type (P<0.01). (C) Normal (I) and nonviable conidia (II) of Δ*mopex19* under light microscopy. Bar = 10 μm. (D) Viable conidia stained with FDA emitted green fluorescence while the dead ones did not. Bar = 10 μm. (E) Statistical analysis of viability of conidia cultured on CM at 28°C for 10 days. Means and standard errors were calculated from three independent repeats (at least 150 conidia of each strain were measured for each repeat). Double asterisks indicate significant differences to the wild-type (P<0.01).

Conidial generation was measured after 10 days culture on CM. Conidiation of the Δ*mopex19* mutants was dramatically reduced by ∼50-fold compared with the wild-type and complemented strains (Fig. 10A and 10B). Cell death was also found in the conidia of Δ*mopex19* mutants (Fig. 10C). Almost all the conidia of the wild-type and complemented strains were adapt to FDA staining and emitted green fluorescence, compared with only ∼30% of those of the Δ*mopex19* mutants, demonstrating reduced conidial vitality in the mutants (Fig. 10D and 10E). These results indicated that *MoPEX19* was required for conidial generation and viability of *M. oryzae*. The cell death in the hyphae and conidia of the mutants may have been trigged by accumulation of cellular ROS [10], consistent with their hypersensitivity to H_2_O_2_ and methyl viologen.

### *MoPEX19* was required for conidial germination and appressorial development and turgor generation

**Figure 11:**
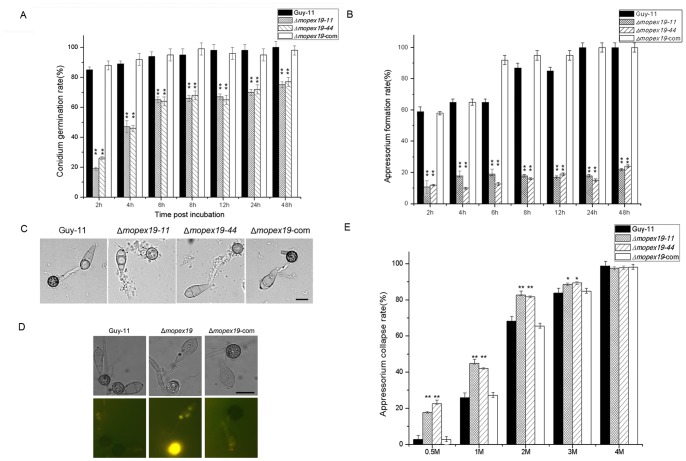
Conidial germination and appressorial formation of Δ*mopex19*. The conidial germination rates (A) and appressorial formation rates (B) of Δ*mopex19*, wild-type and complemented strains on hydrophobic surface were calculated. (C) Conidia and appressoria incubated for 12 h. The appressoria of Δ*mopex19* were less pigmented than those of the control strains, and cellular leakage occurred in the germ tubes and appressoria of Δ*mopex19*. (D) Nile red staining showed that more lipid residues were present in the conidia and germ tubes of Δ*mopex19* incubated for 24 h. (E) Cytorrhysis assay to compare the appressorial turgor genesis of Δ*mopex19* and the controls. The 24-h appressoria were soaked in glycerol and the rates of cytorrhysis were calculated. Means and standard errors were calculated from three independent repeats (at least 100 conidia of each strain were measured for each repeat). Single asterisks indicate significant differences at P<0.05 and double asterisks indicate P<0.01.

The rates of conidial germination and appressorial formation were both significantly reduced in the Δ*mopex19* mutants (Fig. 11A and 11B). Under light microscopy, the 24-h appressoria of Δ*mopex19* mutants were less pigmented than those of the wild-type, corresponding to reduced colonial melanization. Cytoplasmic leakage was found during the germination and appressorial development of the Δ*mopex19* mutants, which arose from the initial germ tubes and increased subsequently (Fig. 11C). This leakage was maybe related to the absence of woronin bodies. Consistent with the defects in lipids metabolism, the lipid mobilization from the conidia to appressoria was blocked in the mutants. Fluorescent microscopy combined with Nile red staining visualized the residual lipids in the conidia of the mutants, whereas hardly in the wild-type conidia incubated for 24 h (Fig. 11D).

The defects in appressorial pigmentation, lipid mobilization to the appressoria, and cytoplasmic leakage may affect substance accumulation and turgor generation in the appressoria. We compared the turgor of Δ*mopex19* mutants and the wild-type and complemented strains by incipient-cytorrhysis technique. Treatment with 1, 2 and 3 M glycerol led to appressorial collapse in the Δ*mopex19* mutants at significantly higher level than that in the wild-type and complemented strains (Fig. 11E). These data indicated that the *MoPEX19* gene plays an important role in appressorial morphogenesis and turgor generation, which are the key factors in host penetration.

### Deletion of *MoPEX19* altered integrity of cell wall

**Figure 12:**
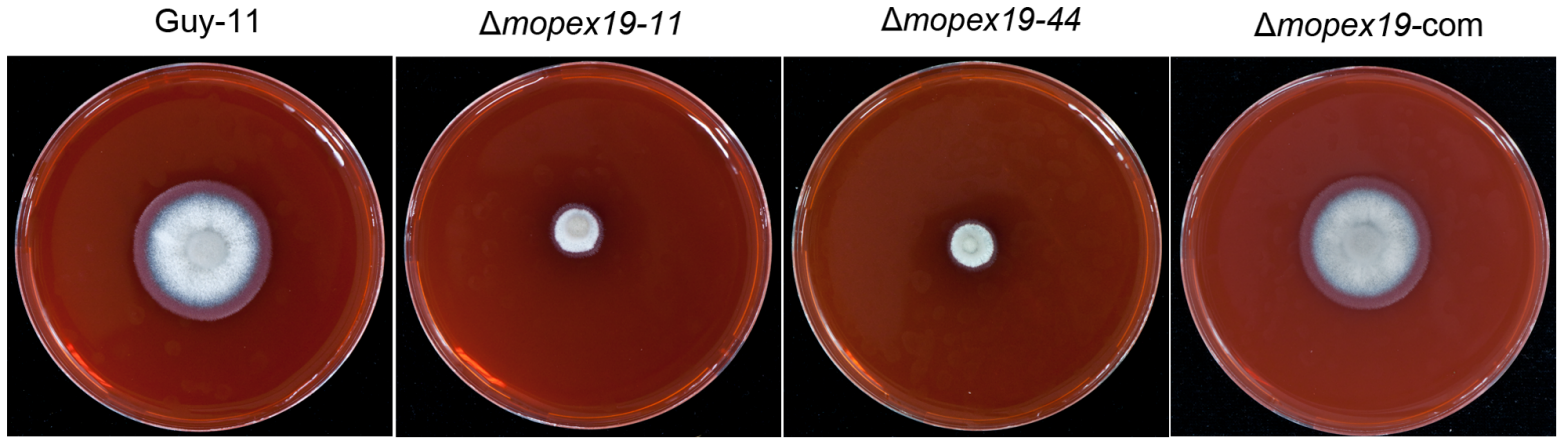
Tolerance to CR to compare the integrity of the cell wall. Wild-type, Δ*mopex19* and complemented strains were cultured on CM supplemented with 200 μg/ml CR for 5 days.

The reduced melanization may weaken cell wall [45] and the cytoplasmic leakage also hints cell wall damage. To confirm whether *MoPEX19* deletion altered the integrity of the cell walls, the tolerance of the Δ*mopex19* mutants to Congo red (CR), a cell wall disrupting agent, was compared with that of the control strains. Cultured on medium supplemented with 200 μg/ml CR, the colonial diameters of the mutants were remarkably lower than those of the wild-type and complemented strains (Fig. 12), indicating that *MoPEX19* played a role in the integrity of cell walls of *M. oryzae*.

### *MoPEX19* is required for pathogenicity

**Figure 13:**
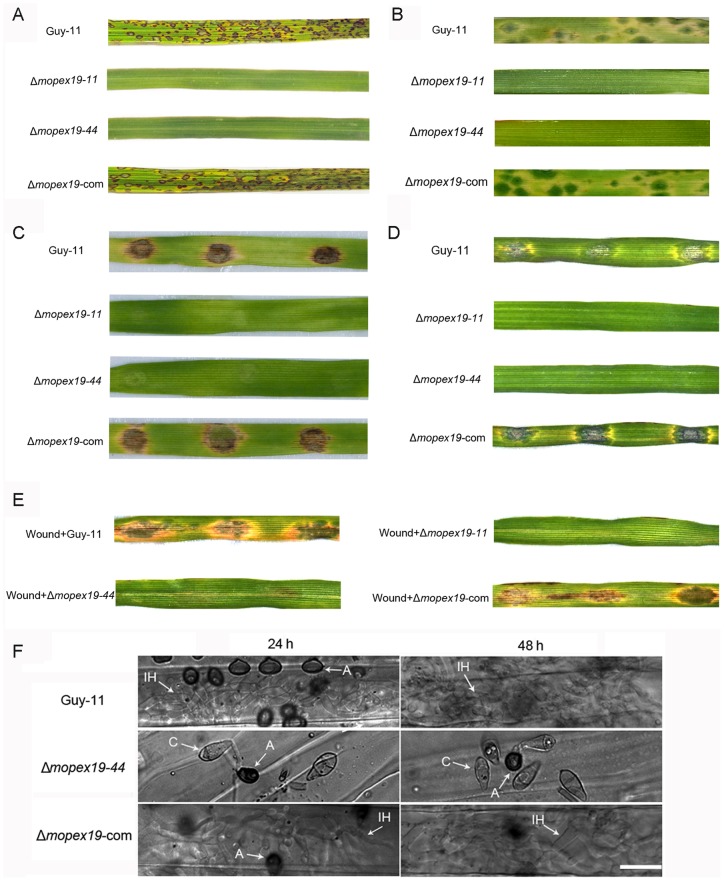
Pathogenicity test of Δ*mopex19*. (A) Spray-inoculation with conidial suspension (1×10^5^ conidia/ml) of Δ*mopex19*, wild-type and complemented strains on 2-week-old rice cultivar CO39. The symptoms were recorded at 7 days post-inoculation. (B) Spray-inoculation with conidial suspension (2×10^4^ conidia/ml) on 7-day-old barley cultivar ZJ-8. The symptoms were recorded at 4 days post-inoculation. Detached barley leaves inoculated with 5-mm mycelial plugs (C) or with 20-μl droplets of conidial suspensions (2×10^4^ conidia/ml) (D) were cultured for 4 days. (E) Wounded barley leaves inoculated with 20-μl conidial suspensions (2×10^4^ conidia/ml) were cultured for 4 days. (F) Droplet-inoculated barley leaves were sampled at 24 and 48 h post-inoculation, discolored, and examined under a light microscope. The conidia (C), appressoria (A) and invasive hyphae (IH) are marked. Bar = 20 μm.

To determine whether the alteration of appressorial melanization, turgor generation and cell wall integrity destroyed pathogenicity of the fungus, we performed inoculation tests on both rice and barley seedlings. The 14-day-old rice cultivar CO39 and 7-day-old barley cultivar ZJ-8 were inoculated by conidial suspensions. No symptoms developed on rice at 7 days post-inoculation or barley leaves at 3 days post-inoculation infected with the Δ*mopex19* mutants, in contrast to those infected with the wild-type and complemented strains, which generated abundant typical lesions (Fig. 13A and 13B). After inoculation with conidial droplets or mycelia plugs, the Δ*mopex19* mutants did not cause any symptoms on detached barley leaves (Fig. 13C and 13D). Even on the wounded leaves from which cuticles were removed, the Δ*mopex19* mutants were still non-pathogenic (Fig. 13E). By histological microscopy, abundant invasive hyphae were visible on the inoculation sites of the wild-type and complemented strains, which expanded quickly in the host tissue. However, no penetration peg or invasive hypha was found on the leaves inoculated with the Δ*mopex19* mutant up to 48 h post-inoculation (Fig. 13F). The results indicated that *MoPEX19* was indispensable to fungal pathogenicity and the *MoPEX19* deletion destroyed host infection of the rice blast fungus completely.

## Discussion

An increasing number of recent studies have revealed the machinery of peroxisomal biogenesis in filamentous fungi, which make it a good model for research in this field [29], [46]. Peroxisomal biogenesis has already been proved to be related to the pathogenicity of plant fungi, such as *M. oryzae*, *C. lagenarium* and *F. graminearum* [9], [10], [30]. However, the previous studies focused only on the peroxins participating in the import of peroxisomal matrix proteins, while the mechanism of PMP import and its roles in fungal pathogenicity remained unclear. Here, we characterized *MoPEX19*, an essential factor in peroxisomal biogenesis, and demonstrated that Mopex19p was indispensable for PMP targeting and maintenance of the peroxisomes, and was required in the pathogenicity and multiple developmental processes of the rice blast fungus *M. oryzae*.

### Role of *MoPEX19* in peroxisomal membrane biogenesis

Although the involvement of *PEX19* in peroxisomal membranes biogenesis was well demonstrated in mammalian and yeast models [21], [47], the functions of *PEX19* are poorly understood in filamentous fungi. In present work, we demonstrated that *PMP47*, a representative PMP, was localized in the peroxisomes of the wild-type strain of *M. oryzae*, but in the cytoplasm of *Δmopex19* mutants, indicating the role of *MoPEX19* in PMP import. In most studies, Pex19p was thought to be a receptor or soluble chaperone that binds the PMPs and maintains their solubility in the cytosol, delivers them to the peroxisomes and facilities their insertion into peroxisomal membranes [48], [49]. In such cases, *PEX19* mutation led to cytosolic localization of PMPs and severe damage, even complete absence, of peroxisomal structures, as that in *pex19* mutants of *S. cerevisiae* and *Hansenula polymorpha* [21], [50], [51]. Consistent with its function as a soluble receptor and/or chaperone, Pex19p was primarily cytosolic and associated with the peroxisomal membrane [18], [22], [52]. However in *Yarrowia lipolytica* and *Pichia pastoris*, Pex19p seemed not to act as a PMP receptor, because Ylpex19p was primarily located in the peroxisomes, and in Δ*ylpex19* and Δ*Pppex19* cells, peroxisome remnants were found and PMPs were detected on these peroxisome-resembling structures [53], [54]. In Δ*mopex19* mutants, no peroxisome or peroxisome-like structures were detected, suggesting that *MoPEX19* was indispensable for peroxisomal maintenance. Resulted from the peroxisomal disappearance, the peroxisomal matrix proteins (PTS1- and PTS2-containing proteins) were distributed cytosolically in Δ*mopex19* mutants, as the situation in *pex19* mutants of *S. cerevisiae* and mammalians [21], [24]. Mopex19p was found mainly in the cytosol, with punctate enhancements that were possibly newly formed peroxisomes. *MoPEX19* recovered the ability of Δ*scpex*19 mutant to utilize lipids. These findings suggest that *MoPEX19* has a similar function to that in *S. cerevisiae* rather than in *Y. lipolytica* or *P. pastoris*. Furthermore, the imperfect overlap of GFP–MoPEX19 with RFP–PTS1 also agreed with the function of *MoPEX19* in PMP import, because PMP assembly usually occurred before the import of peroxisomal matrix proteins represented by RFP–PTS1 [54]. Taken together, our data confirm that Mopex19 acts as a PMP receptor or chaperone that plays key roles in peroxisomal membrane assembly and maintenance of peroxisomal structures in *M. oryzae*.

The defects of *P. pastoris pex19* mutant were functionally complemented by *H. polymorpha PEX19* [51]. The human *HsPEX19* recovered the import of peroxisomal matrix proteins and PMPs in *pex19*-mutated Chinese hamster ovary cells [24]. However, the Δ*scpex19* mutant was not complemented by intact human *PEX19* gene [21]. These results indicated that *PEX19* is functionally interchangeable between closely related species but not conserved between yeasts and mammals. Our data showed that *MoPEX19* complemented the growth defects of the Δ*scpex19* mutant on oleic acid, implying that the functional conservation of Pex19p can span the evolutionary distance between filamentous fungi and yeasts. Human Pex19p acquired the complementary ability to Δ*scpex19* mutant when its N-terminal region is substituted by that of Scpex19p, indicating that the N- and C-terminal regions of Pex19p have distinct functions and exhibited different conservation between organisms [21]. It will therefore be interesting to investigate the functions and conservations of different parts of Mopex19p in future.

### Influence of *MoPEX19* on peroxisomal metabolism and fungal development

Peroxisomes contain >50 enzymes involved in diverse metabolic processes, typically, fatty acid β-oxidation, glyoxylate cycle, and H_2_O_2_ elimination [1]. β-Oxidation is confined to the peroxisomes in yeast and plants, whereas it is present in both peroxisomes and mitochondria in mammalian cells [55]–[57]. In filamentous fungi, β-oxidation had been traditionally thought to occur exclusively in peroxisomes, until the discovery of mitochondrial β-oxidation led to acceptance of diverse β-oxidation pathways [58]–[60]. Δ*mopex19* could not grow on fatty acids as sole carbon source, same as described previously in Δ*mopex5* and Δ*mopex6* [8], [30], [33], suggesting strongly that fatty acid β-oxidation in *M. oryzae* takes place predominately or exclusively in peroxisomes. Similarly, disruption of *PEX6* disrupted growth of *C. lagenarium* on oleic acid, and *pex* mutants of *A. nidulans* exhibited severe reduction in fatty acid utilization [9], [15]. However in *F. graminearum*, fatty acid utilization was slightly affected by *PEX5* and *PEX6* deletion, reflecting the offset of mitochondrial β-oxidation [10]. These results indicate that peroxisomal and mitochondrial β-oxidation are both present in filamentous fungi and the proportions of the two metabolic pathways are species specific.

Most of the enzymes involved in the glyoxylate cycle are located in peroxisomes in yeasts and mammals [61], [62]. The Δ*mopex19* mutants lost the ability to utilize acetate, reflecting disorders in the glyoxylate cycle and suggesting that the cycle in *M. oryzae* occurs in peroxisomes. This agrees with previous studies on Icl1p, a core enzyme in the glyoxylate cycle. Icl1p was localized in the peroxisomes in *C. lagenarium*, and disruption of *ICL1* gene led to the incapability of *M. oryzae* and *C. lagenarium* to grow on acetate [63], [64]. Furthermore, deletion of carnitine aceryltransterase, which mediates the movement of acetyl-CoA from cytoplasm to peroxisome, also prevented acetate utilization in *M. oryzae*. Surprisingly, the mutants *MoPEX5*, *MoPEX6* and *MoPEX7* all grow normally on acetate [8], [30], [32], [33]. This may suggest the presence of novel import machinery for matrix proteins, which translocates the enzymes for the glyoxylate cycle into peroxisomes in a manner independent of *PEX5*, *PEX6* and *PEX7*. Consistent with this hypothesis, neither typical PTS1 nor PTS2 are found in Icl1p [64].

The end product of fatty acid β-oxidation, acetyl-CoA, together with glycolysis end products, can serve as energy donors that enter the citric acid cycle to produce ATP, or as constituents for synthesis of cellular components. Acetyl-CoA can be metabolized via the gluconeogenesis pathway to generate glycerol, which is the main solute in appressoria and indispensable for turgor generation [39]. The appressorial turgor of Δ*mopex19* was dramatically reduced, as well in other *pex* mutants of *M. oryzae* and *C. lagenarium* [8], [30], [32], [33], [42], indicating that the acetyl-CoA derived from β-oxidation contributes largely to appressorial glycerol generation. Acetyl-CoA can also be a starter unit of melanin, which could be the reason for why Δ*mopex19* mutants showed decreased melanization in colony and appressoria. The shortage of appressorial melanization, which is crucial for maintenance of the cell wall strength and impermeability to glycerol [65], led to severe cellular leakage in germ tubes and appressoria of Δ*mopex19*. Thus, the decrease in appressorial turgor is a synergy of decreased synthesis and leakage of glycerol, and the two aspects could result from the shortage of acetyl-CoA caused by insufficient fatty acid conversion. In addition, the cellular leakage reminds us of the function of woronin bodies, peroxisome-derived organelles that plug the septum pores when hyphae are damaged [34]. As expected, the woronin bodies could not be detected in Δ*mopex19*.

Deficient fatty acid β-oxidation resulted in disordered appressorial turgor generation, which was crucial for fungal penetration. However, it seems not to be the sole reason leading to failure of infection with Δ*mopex19*, because adding exogenous carbon did not recover the pathogenicity. Accordingly, only partial recovery of pathogenicity was found in other *pex* mutants by adding glucose or intermediate compounds of β-oxidation and glyoxylate cycle [8]. In *F. graminearum*, which does not form appressoria, the *PEX* genes still play a crucial role in pathogenicity [10]. All these facts hint that other major metabolic pathways in peroxisomes contribute to the fungal pathogenicity. ROS scavenging is another peroxisomal metabolism and the peroxisomal disorder enhanced ROS accumulation in yeast [66]–[68]. A peroxisome-associated protein, TmpL, is involved in intracellular ROS regulation in *Alternaria brassicicola* and *A. fumigates* [69]. In Δ*pex5* cells of *F. graminearum* and *M. oryzae*, ROS tolerance was reduced and cell death occurred [10], [33]. In present work, the growth of Δ*mopex19* was markedly reduced in ROS-containing media. The viability of its mycelia and asexual spores decreased, as seen in Δ*pex5* and Δ*pex6* mutants of *M. oryzae* and *F. graminearum*, which was thought to be trigged by redundant cellular oxidative stress [10], [70]. These results demonstrate that the involvement of *MoPEX19* in ROS scavenging. However, mitochondria are also major organelles for ROS metabolism, which were demonstrated crucial in ROS neutralization and post-penetration growth in *M. oryzae* [71]. Disruption mitochondrial enoyl-CoA hydratase, Ech1, enhanced the sensitivity to oxidative stress and reduced the host penetration of the fungus. The peroxisomes and mitochondria are thus both required in regulating the ROS levels in filamentous fungi. The reduction of ROS scavenging in Δ*mopex19* is likely another reason for its infection failure, because ROS produced by the host cells is a main barrier for fungal invasion and establishment of parasitism.

In *M. oryzae*, three *PEX* genes have been characterized to date: *MoPEX5*, *MoPEX6* and *MoPEX7*. *MoPEX5* contributes to import of PTS1 matrix protein; *MoPEX7* is involved in PTS2 matrix protein import; and *MoPEX6* participates in both PTS1 and PTS2 import [8], [30], [32], [33]. Here, we demonstrated that *MoPEX19* contributed most among the three genes to peroxisomal biogenesis, because it influenced the import of both matrix proteins and PMPs. This led us to propose that *MoPEX19* plays more roles in peroxisomal metabolism, fungal development, and host invasion. According to our data, more severe metabolic defects were found in the Δ*mopex19* mutants, such as the incapacity of acetate utilization and cellular leakage, which were not observed in the other reported pex mutants. However, the development and pathogenicity of Δ*mopex19* did not differ markedly to those of Δ*mopex5* and Δ*mopex6* [8], [30], [33]. This confirmed our previous conclusion that PTS1 contributes predominantly to fungal development and pathogenicity [33]. The peroxisomes play essential roles in the sexual reproduction of *F. graminearum* and *Podospora anserina* [10], [26], but the sexual generation is unaffected in Δ*mopex19* and any other *pex* mutant of *M. oryzae* [8], [30], [32], [33]. This reflects the dissimilarity of the roles played by peroxisomal metabolism between fungal species.

In summary, as a key factor in peroxisomal biogenesis, *MoPEX19* participated in import of PMPs. Deletion of *MoPEX19* blocked the transport of PMPs from cytoplasm to peroxisomes, which led to complete disappearance of peroxisomal structures and improper localization of peroxisomal matrix proteins. The disorder in peroxisomal biogenesis disturbed severely peroxisomal metabolism, including fatty acid β-oxidation, the glyoxylate cycle, melanization, and cell wall synthesis. The metabolic defects led to multiple abnormalities of fungal development and resulted in loss of pathogenicity.

## References

[1] Wanders RJA (2004) Peroxisomes, lipid metabolism, and peroxisomal disorders. Molecular Genetics and Metabolism 83: 16–27. 15464416 10.1016/j.ymgme.2004.08.016

[2] Faust PL, Banka D, Siriratsivawong R, Ng VG, Wikander TM (2005) Peroxisome biogenesis disorders: The role of peroxisomes and metabolic dysfunction in developing brain. Journal of Inherited Metabolic Disease 28: 369–383. 15868469 10.1007/s10545-005-7059-y

[3] Wanders RJA, Waterham HR (2005) Peroxisomal disorders I: biochemistry and genetics of peroxisome biogenesis disorders. Clinical Genetics 67: 107–133. 15679822 10.1111/j.1399-0004.2004.00329.x

[4] Holroyd C, Erdmann R (2001) Protein translocation machineries of peroxisomes. FEBS Letters 501: 6–10. 11457447 10.1016/s0014-5793(01)02617-5

[5] Purdue PE, Lazarow PB (2001) Peroxisome biogenesis. Annual Review of Cell and Developmental Biology 17: 701–752. 10.1146/annurev.cellbio.17.1.701 11687502

[6] Distel B, Erdmann R, Gould SJ, Blobel G, Crane DI, et al (1996) Unified nomenclature for peroxisome biogenesis factors. Journal of Cell Biology 135: 1–3. 8858157 10.1083/jcb.135.1.1 PMC2121017

[7] Kiel J, Veenhuis M, van der Klei IJ (2006) PEX genes in fungal genomes: Common, rare or redundant. Traffic 7: 1291–1303. 16978390 10.1111/j.1600-0854.2006.00479.x

[8] Ramos-Pamplona M, Naqvi NI (2006) Host invasion during rice-blast disease requires carnitine-dependent transport of peroxisomal acetyl-CoA. Molecular Microbiology 61: 61–75. 16824095 10.1111/j.1365-2958.2006.05194.x

[9] Kimura A, Takano Y, Furusawa I, Okuno T (2001) Peroxisomal metabolic function is required for appressorium-mediated plant infection by *Colletotrichum lagenarium* . Plant Cell 13: 1945–1957. 11487704 10.1105/TPC.010084 PMC139132

[10] Min K, Son H, Lee J, Choi GJ, Kim JC, et al (2012) Peroxisome function is required for virulence and survival of *Fusarium graminearum* . Molecular Plant-Microbe Interactions 25: 1617–1627. 22913493 10.1094/MPMI-06-12-0149-R

[11] Marzioch M, Erdmann R, Veenhuis M, Kunau WH (1994) PAS7 encodes a novel yeast member of the WD-40 protein family essential for import of 3-oxoacyl-coa thiolase, a PTS2-containing protein, into peroxisomes. EMBO Journal 13: 4908–4918. 7957058 10.1002/j.1460-2075.1994.tb06818.x PMC395431

[12] Dodt G, Braverman N, Valle D, Gould SJ (1996) From expressed sequence tags to peroxisome biogenesis disorder genes. Peroxisomes: Biology and Role in Toxicology and Disease 804: 516–523. 10.1111/j.1749-6632.1996.tb18641.x 8993569

[13] Elgersma Y, Kwast L, Klein A, VoornBrouwer T, vandenBerg M, et al (1996) The SH3 domain of the *Saccharomyces cerevisiae* peroxisomal membrane protein Pex13p functions as a docking site for Pex5p, a mobile receptor for the import of PTS1-containing proteins. Journal of Cell Biology 135: 97–109. 8858166 10.1083/jcb.135.1.97 PMC2121018

[14] Rehling P, Albertini M, Kunau WH (1996) Protein import into peroxisomes: New developments. Peroxisomes: Biology and Role in Toxicology and Disease 804: 34–46. 10.1111/j.1749-6632.1996.tb18606.x 8993534

[15] Hynes MJ, Murray SL, Khew GS, Davis MA (2008) Genetic analysis of the role of peroxisomes in the utilization of acetate and fatty acids in *Aspergillus nidulans* . Genetics 178: 1355–1369. 18245820 10.1534/genetics.107.085795 PMC2278063

[16] Wang JY, Wu XY, Zhang Z, Du XF, Chai RY, et al (2008) Fluorescent co-localization of PTS1 and PTS2 and its application in analysis of the gene function and the peroxisomal dynamic in *Magnaporthe oryzae* . Journal of Zhejiang University Sciences B 9: 802–810. 10.1631/jzus.B0860001 PMC2565744 18837108

[17] Girzalsky W, Saffian D, Erdmann R (2010) Peroxisomal protein translocation. Biochimica Et Biophysica Acta 1803. 10.1016/j.bbamcr.2010.01.002 20079383

[18] Fransen M, Wylin T, Brees C, Mannaerts GP, Van Veldhoven PP (2001) Human Pex19p binds peroxisomal integral membrane proteins at regions distinct from their sorting sequences. Molecular and Cellular Biology 21: 4413–4424. 11390669 10.1128/MCB.21.13.4413-4424.2001 PMC87101

[19] Jones AB (2001) Peroxisome proliferator-activated receptor (PPAR) modulators: Diabetes and beyond. Medicinal Research Reviews 21: 540–552. 11607934 10.1002/med.1025

[20] Rottensteiner H, Kramer A, Lorenzen S, Stein K, Landgraf C, et al (2004) Peroxisomal membrane proteins contain common Pex19p-binding sites that are an integral part of their targeting signals. Molecular Biology of the Cell 15: 3406–3417. 15133130 10.1091/mbc.E04-03-0188 PMC452593

[21] Gotte K, Girzalsky W, Linkert M, Baumgart E, Kammerer S, et al (1998) Pex19p, a farnesylated protein essential for peroxisome biogenesis. Molecular and Cellular Biology 18: 616–628. 9418908 10.1128/mcb.18.1.616 PMC121529

[22] Sacksteder KA, Jones JM, South ST, Li X, Liu Y, et al (2000) *PEX19* binds multiple peroxisomal membrane proteins, is predominantly cytoplasmic, and is required for peroxisome membrane synthesis. The Journal of Cell Biology 148: 931–944. 10704444 10.1083/jcb.148.5.931 PMC2174547

[23] Shibata N, Tsunekawa N, Okamoto-Ito S, Akasu R, Tokumasu A, et al (2004) Mouse RanBPM is a partner gene to a germline specific RNA helicase, mouse vasa homolog protein. Molecular Reproduction and Development 67: 1–7. 14648869 10.1002/mrd.20009

[24] Matsuzono Y, Kinoshita N, Tamura S, Shimozawa N, Hamasaki M, et al (1999) Human PEX19: cDNA cloning by functional complementation, mutation analysis in a patient with Zellweger syndrome, and potential role in peroxisomal membrane assembly. Proceedings of the National Academy of Sciences of the United States of America 96: 2116–2121. 10051604 10.1073/pnas.96.5.2116 PMC26746

[25] Reiss J (1971) Cytochemical localization of peroxisomes in fungal cells. Protoplasma 72: 43–48. 5578690 10.1007/BF01281009

[26] Bonnet C, Espagne E, Zickler D, Boisnard S, Bourdais A, et al (2006) The peroxisomal import proteins *PEX2*, *PEX5* and *PEX7* are differently involved in *Podospora anserina* sexual cycle. Molecular Microbiology 62: 157–169. 16987176 10.1111/j.1365-2958.2006.05353.x

[27] Hynes MJ, Murray SL, Duncan A, Khew GS, Davis MA (2006) Regulatory genes controlling fatty acid catabolism and peroxisomal functions in the filamentous fungus *Aspergillus nidulans* . Eukaryotic Cell 5: 794–805. 16682457 10.1128/EC.5.5.794-805.2006 PMC1459687

[28] Managadze D, Wurtz C, Sichting M, Niehaus G, Veenhuis M, et al (2007) The peroxin *PEX14* of *Neurospora crassa* is essential for the biogenesis of both glyoxysomes and Woronin bodies. Traffic 8: 687–701. 17461798 10.1111/j.1600-0854.2007.00560.x

[29] Idnurm A, Giles SS, Perfect JR, Heitman J (2007) Peroxisome function regulates growth on glucose in the basidiomycete fungus *Cryptococcus neoformans* . Eukaryotic Cell 6: 60–72. 17041184 10.1128/EC.00214-06 PMC1800366

[30] Wang ZY, Soanes DM, Kershaw MJ, Talbot NJ (2007) Functional analysis of lipid metabolism in *Magnaporthe grisea* reveals a requirement for peroxisomal fatty acid beta-oxidation during appressorium-mediated plant infection. Molecular Plant-Microbe Interactions 20: 475–491. 17506326 10.1094/MPMI-20-5-0475

[31] Fujihara N, Sakaguchi A, Tanaka S, Fujii S, Tsuji G, et al (2010) Peroxisome biogenesis factor *PEX13* is required for appressorium-mediated plant infection by the anthracnose fungus *Colletotrichum orbiculare* . Molecular Plant-Microbe Interactions 23: 436–445. 20192831 10.1094/MPMI-23-4-0436

[32] Goh J, Jeon J, Kim KS, Park J, Park S-Y, et al (2011) The *PEX7*-mediated peroxisomal import system is required for fungal development and pathogenicity in *Magnaporthe oryzae* . PLoS One 6: e28220. 22194815 10.1371/journal.pone.0028220 PMC3237427

[33] Wang JY, Zhang Z, Wang YL, Li L, Chai RY, et al (2013) PTS1 peroxisomal import pathway plays shared and distinct roles to PTS2 pathway in development and pathogenicity of *Magnaporthe oryzae* . PLoS One 8: e55554. 23405169 10.1371/journal.pone.0055554 PMC3566003

[34] Soundararajan S, Jedd G, Li X, Ramos-Pamplona M, Chua NH, et al (2004) Woronin body function in *Magnaporthe grisea* is essential for efficient pathogenesis and for survival during nitrogen starvation stress. Plant Cell 16: 1564–1574. 15155882 10.1105/tpc.020677 PMC490046

[35] Valent B (1990) Rice blast as a model system for plant pathology. Phytopathology 80: 33–36.

[36] Hamer JE, Howard RJ, Chumley FG, Valent B (1988) A mechanism for surface attachment in spores of a plant pathogenic fungus. Science 239: 288–290. 17769992 10.1126/science.239.4837.288

[37] Howard RJ, Valent B (1996) Breaking and entering: Host penetration by the fungal rice blast pathogen *Magnaporthe grisea* . Annual Review of Microbiology 50: 491–512. 10.1146/annurev.micro.50.1.491 8905089

[38] Howard RJ, Ferrari MA, Roach DH, Money NP (1991) Penetration of hard substrates by a fungus employing enormous turgor pressures. Proc Natl Acad Sci U S A 88: 11281–11284. 1837147 10.1073/pnas.88.24.11281 PMC53118

[39] de Jong JC, McCormack BJ, Smirnoff N, Talbot NJ (1997) Glycerol generates turgor in rice blast. Nature 389: 244–244.

[40] Notteghem JL, Silue D (1992) Distribution of the mating type alleles in *Magnaporthe grisea* populations pathogenic on rice. Phytopathology 82: 421–424.

[41] Talbot NJ, Ebbole DJ, Hamer JE (1993) Identification and characterization of *MPG1*, a gene involved in pathogenicity from the rice blast fungus *Magnaporthe grisea* . Plant Cell 5: 1575–1590. 8312740 10.1105/tpc.5.11.1575 PMC160387

[42] Crawford MS, Chumley FG, Weaver CG, Valent B (1986) Characterization of the heterokaryotic and vegetative diploid Phases of *Magnaporthe grisae* . Genetics 114: 1111–1129. 17246357 10.1093/genetics/114.4.1111 PMC1203031

[43] Rho HS, Kang S, Lee YH (2001) *Agrobacterium tumefaciens*-mediated transformation of the plant pathogenic fungus, *Magnaporthe grisea* . Molecules and Cells 12: 407–411. 11804343

[44] Liu XH, Lu JP, Zhang L, Dong B, Min H, et al (2007) Involvement of a Magnaporthe grisea serine/threonine kinase gene, MgATG1, in appressorium turgor and pathogenesis. Eukaryot Cell 6: 997–1005. 17416896 10.1128/EC.00011-07 PMC1951528

[45] Pihet M, Vandeputte P, Tronchin G, Renier G, Saulnier P, et al (2009) Melanin is an essential component for the integrity of the cell wall of *Aspergillus fumigatus* conidia. BMC Microbiol 9: 177. 19703288 10.1186/1471-2180-9-177 PMC2740851

[46] Sichting M, Schell-Steven A, Prokisch H, Erdmann R, Rottensteiner H (2003) Pex7p and Pex20p of *Neurospora crassa* function together in PTS2-dependent protein import into peroxisomes. Molecular Biology of the Cell 14: 810–821. 12589072 10.1091/mbc.E02-08-0539 PMC150010

[47] Nuttall JM, Motley A, Hettema EH (2011) Peroxisome biogenesis: recent advances. Current Opinion in Cell Biology 23: 421–426. 21689915 10.1016/j.ceb.2011.05.005

[48] Shibata H, Kashiwayama Y, Imanaka T, Kato H (2004) Domain architecture and activity of human Pex19p, a chaperone-like protein for intracellular trafficking of peroxisomal membrane proteins. J Biol Chem 279: 38486–38494. 15252024 10.1074/jbc.M402204200

[49] Fang Y, Morrell JC, Jones JM, Gould SJ (2004) *PEX3* functions as a *PEX19* docking factor in the import of class I peroxisomal membrane proteins. The Journal of Cell Biology 164: 863–875. 15007061 10.1083/jcb.200311131 PMC2172291

[50] Hettema EH, Girzalsky W, van Den Berg M, Erdmann R, Distel B (2000) *Saccharomyces cerevisiae* pex3p and pex19p are required for proper localization and stability of peroxisomal membrane proteins. The EMBO Journal 19: 223–233. 10637226 10.1093/emboj/19.2.223 PMC305556

[51] Otzen M, Perband U, Wang D, Baerends RJ, Kunau WH, et al (2004) *Hansenula polymorpha* Pex19p is essential for the formation of functional peroxisomal membranes. J Biol Chem 279: 19181–19190. 14981078 10.1074/jbc.M314275200

[52] Fransen M, Vastiau I, Brees C, Brys V, Mannaerts GP, et al (2005) Analysis of human Pex19p's domain structure by pentapeptide scanning mutagenesis. Journal of Molecular Biology 346: 1275–1286. 15713480 10.1016/j.jmb.2005.01.013

[53] Lambkin GR, Rachubinski RA (2001) *Yarrowia lipolytica* cells mutant for the peroxisomal peroxin Pex19p contain structures resembling wild-type peroxisomes. Molecular Biology of the Cell 12: 3353–3364. 11694572 10.1091/mbc.12.11.3353 PMC60260

[54] Snyder WB, Koller A, Choy AJ, Subramani S (2000) The peroxin Pex19p interacts with multiple, integral membrane proteins at the peroxisomal membrane. Journal of Cell Biology 149: 1171–1177. 10851015 10.1083/jcb.149.6.1171 PMC2175117

[55] Beevers H (1982) Glyoxysomes in higher plants. Annals of the New York Academy of Sciences 386: 243–251.

[56] Schulz H (1991) Beta oxidation of fatty acids. Biochimica et Biophysica Acta 1081: 109–120. 1998729 10.1016/0005-2760(91)90015-a

[57] Kunau WH, Dommes V, Schulz H (1995) beta-oxidation of fatty acids in mitochondria, peroxisomes, and bacteria: a century of continued progress. Progress in Lipid Research 34: 267–342. 8685242 10.1016/0163-7827(95)00011-9

[58] Valenciano S, DeLucas JR, Pedregosa A, Monistrol IF, Laborda F (1996) Induction of beta-oxidation enzymes and microbody proliferation in *Aspergillus nidulans* . Archives of Microbiology 166: 336–341. 8929280 10.1007/s002030050392

[59] Maggio – Hall LA, Keller NP (2004) Mitochondrial β – oxidation in *Aspergillus nidulans* . Molecular Microbiology 54: 1173–1185. 15554960 10.1111/j.1365-2958.2004.04340.x

[60] Shen YQ, Burger G (2009) Plasticity of a key metabolic pathway in fungi. Functional & Integrative Genomics 9: 145–151. 18795352 10.1007/s10142-008-0095-6

[61] Titorenko VI, Rachubinski RA (2001) Dynamics of peroxisome assembly and function. Trends in Cell Biology 11: 22–29. 11146295 10.1016/s0962-8924(00)01865-1

[62] Titorenko VI, Rachubinski RA (2004) The peroxisome: orchestrating important developmental decisions from inside the cell. Journal of Cell Biology 164: 641–645. 14981090 10.1083/jcb.200312081 PMC2172158

[63] Wang ZY, Thornton CR, Kershaw MJ, Debao L, Talbot NJ (2003) The glyoxylate cycle is required for temporal regulation of virulence by the plant pathogenic fungus *Magnaporthe grisea* . Molecular Microbiology 47: 1601–1612. 12622815 10.1046/j.1365-2958.2003.03412.x

[64] Asakura M, Okuno T, Takano Y (2006) Multiple contributions of peroxisomal metabolic function to fungal pathogenicity in *Colletotrichum lagenarium* . Applied and Environmental Microbiology 72: 6345–6354. 16957261 10.1128/AEM.00988-06 PMC1563638

[65] Money NP, Howard RJ (1996) Confirmation of a link between fungal pigmentation, turgor pressure, and pathogenicity using a new method of turgor measurement. Fungal Genetics and Biology 20: 217–227.

[66] Bonekamp NA, Volkl A, Fahimi HD, Schrader M (2009) Reactive oxygen species and peroxisomes: Struggling for balance. Biofactors 35: 346–355. 19459143 10.1002/biof.48

[67] Legakis JE, Koepke JI, Jedeszko C, Barlaskar F, Terlecky LJ, et al (2002) Peroxisome senescence in human fibroblasts. Mol Biol Cell 13: 4243–4255. 12475949 10.1091/mbc.E02-06-0322 PMC138630

[68] Jungwirth H, Ring J, Mayer T, Schauer A, Buttner S, et al (2008) Loss of peroxisome function triggers necrosis. FEBS Lett 582: 2882–2886. 18656474 10.1016/j.febslet.2008.07.023

[69] Kim K-H, Willger SD, Park S-W, Puttikamonkul S, Grahl N, et al (2009) TmpL, a transmembrane protein required for intracellular redox homeostasis and virulence in a plant and an animal fungal pathogen. PLoS Pathog 5: e1000653. 19893627 10.1371/journal.ppat.1000653 PMC2766074

[70] Sun CB, Suresh A, Deng YZ, Naqvi NI (2006) A multidrug resistance transporter in *Magnaporthe* is required for host penetration and for survival during oxidative stress. Plant Cell 18: 3686–3705. 17189344 10.1105/tpc.105.037861 PMC1785395

[71] Patkar RN, Ramos-Pamplona M, Gupta AP, Fan Y, Naqvi NI (2012) Mitochondrial beta-oxidation regulates organellar integrity and is necessary for conidial germination and invasive growth in Magnaporthe oryzae. Mol Microbiol 86: 1345–1363. 23043393 10.1111/mmi.12060

